# Supplementary material for: Generation of patterned kidney organoids that recapitulate the adult kidney collecting duct system from expandable ureteric bud progenitors
Source: Nat Commun. 2021 Jun 15;12:3641. doi: 10.1038/s41467-021-23911-5 (PMC8206157; doi:10.1038/s41467-021-23911-5)
Supplement: Supplementary file 1 — Supplementary Information [file 41467_2021_23911_MOESM1_ESM.pdf]

Supplementary Information

Generation of Patterned Kidney Organoids that Recapitulate the Adult Kidney Collecting Duct System from Expandable Ureteric Bud Progenitors

Zipeng Zeng[1,2,#], Biao Huang[1,2,#], Riana K. Parvez[2], Yidan Li[1,2], Jyunhao Chen[1,2], Ariel C. Vonk[1,2], Matthew E. Thornton[3], Tadrushi Patel[1,2], Elisabeth A. Rutledge[2], Albert D. Kim[2], Jingying Yu[1,2], Brendan H. Grubbs[3], Jill A. McMahon[2], Nuria M. Pastor-Soler[1], Kenneth R. Hallows[1], Andrew P. McMahon[2,], and Zhongwei Li[1,2*]

Inventory of Supplemental Information

I Supplementary Figures

- Supplementary Fig. 1
- Supplementary Fig. 2
- Supplementary Fig. 3
- Supplementary Fig. 4
- Supplementary Fig. 5
- Supplementary Fig. 6
- Supplementary Fig. 7
- Supplementary Fig. 8

II Supplementary Tables

- Supplementary Table 1
- Supplementary Table 2
- Supplementary Table 3
- Supplementary Table 4
- Supplementary Table 5
- Supplementary Table 6
- Supplementary Table 7
- Supplementary Table 8
- Supplementary Table 9

III Supplementary Methods

IV Supplementary References

Zeng *et al.*, Supplementary Figure 1

a

Stage I Starting from Yuri *et al.*, 2017 using *Wnt11*-RFP

- ✓ Branching morphogenesis confirmed
- ✓ Limited expansion and quick loss of *Wnt11*-RFP

Stage II Optimization of individual components from Yuri *et al.*, 2017

- ✓ Is every component (FGF1, RA, C1, GDNF) essential? ⟶ YES
- ✓ Optimization: RA ⟶ TTNPB
- ✓ Optimization: FGF1 ⟶ FGF9
- ✓ Optimization: C1 ⟶ C3

Stage III Screening of new growth factors and chemicals

- ✓ Base condition (FGF9, TT, C3, GDNF)
- ✓ 1st round screening for individual hits
- ✓ 2nd round screening for combinatorial effects
- ✓ UBCM identified (FGF9, TT, C3, GDNF, LDN, A83, JAKI, SB, Rspo1)

Stage IV Validation of UBCM

- ✓ Is every component essential in the UBCM? ⟶ YES

b

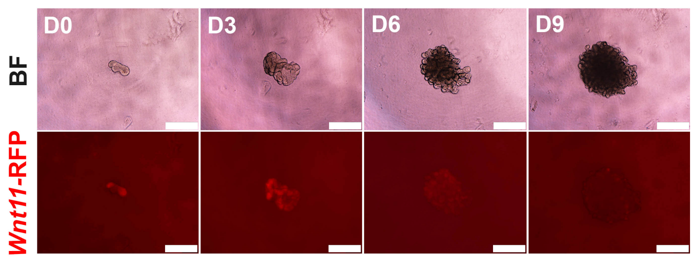

c

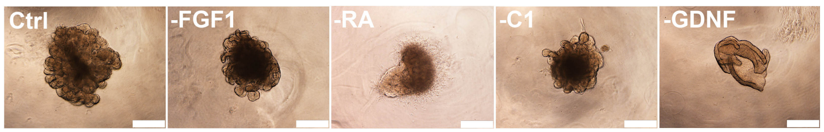

d

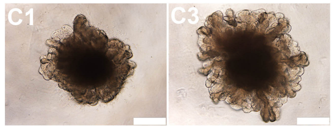

e

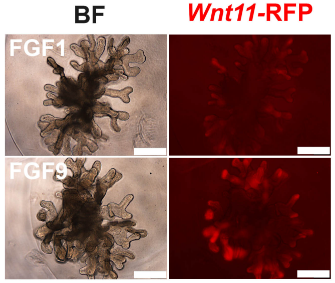

f

Reagent Name	Concentration	*Wnt11*-RFP	Organoid growth	Selected for R2 screening
CHIR99021	6μM	-	-	-
FGF2	200ng/ml	Worse	-	-
FGF4	50ng/ml	Worse	-	-
FGF7	50ng/ml	-	-	Y
FGF8	100ng/ml	Worse	-	-
FGF10	100ng/ml	Worse	Slower	-
FGF20	50ng/ml	Worse	-	-
Activin A	20ng/ml	Worse	Slower	-
A83-01	200nM	Better	-	Y
BMP7	10ng/ml	Better	-	Y
LDN193189	100nM	Better	-	Y
Purmorphamine	1μM	-	-	-
KAAD-Cyclopamine	100nM	-	-	-
JAG-1	1μM	-	-	-
DAPT	200nM	Better	-	Y
SP600126	10μM	-	-	-
SB202190	5μM	Slightly Better	-	Y
PD0325901	1μM	Dead at D6	No growth	-
VEGF	50ng/ml	-	-	-
Y27632	10μM	-	-	-

g

Reagent Name	Concentration	*Wnt11*-RFP	Organoid growth	Selected for R2 screening
R-Spondin 1	100ng/ml	Better	-	Y
Heparin	1μg/ml	Slightly Better	-	Y
SCF	50ng/ml	Better	Slightly better	Y
KSR	15%	Better	-	Y
LY294002	5μM	Better	-	Y
Cyclosporine	10μM	Better	-	Y
TNF-α	100ng/ml	Worse	-	-
HGF	50ng/ml	-	-	-
EGF	50ng/ml	Better	-	Y
Forskolin	10μM	Worse	-	-
LIF	10^3 units/ml	-	-	-
JAKI	100nM	Slightly Better	-	Y
IGF1	20ng/ml	-	-	-
IGF2	2ng/ml	-	-	-
AICAR	0.5mM	Slightly Better	-	Y
Metformin	1mM	Slightly Better	-	Y
Abbott	0.1mM	-	-	-
XMU-MP-1	1μM	-	No growth	-
Verteporfin	1μM	-	Dead	-
PDGF-BB	10ng/ml	-	-	-

h

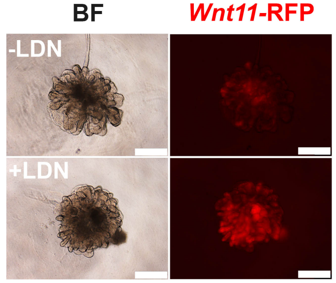

i

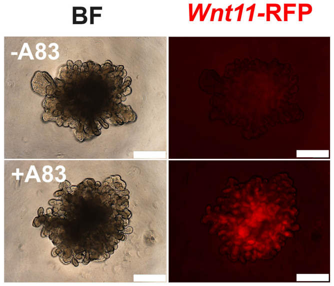

j

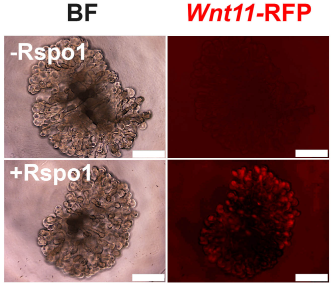

k

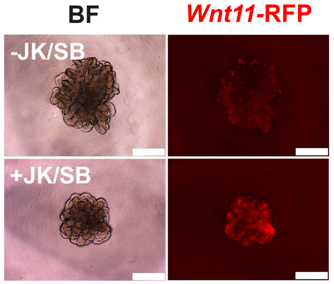

l

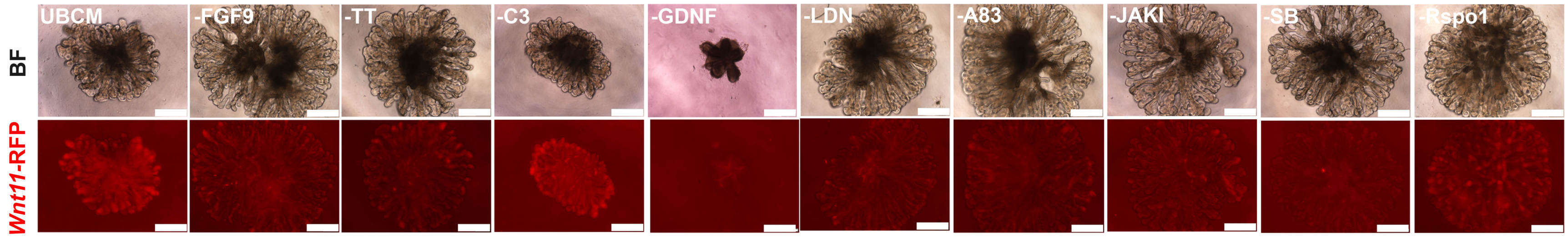

Supplementary Figure 1. Screening for optimal UB culture condition. a, Summary of UB culture condition screening steps. See Supplementary Methods for more details. **b**, Bright field (BF) and fluorescence (*Wnt11*-RFP) images of E11.5 *Wnt11*-RFP UB cultured in previously described UB culture condition[1], from day 0 (D0), day 3 (D3), day 6 (D6) to day 9 (D9). Scale bars, 200 μm. **c**, Bright field images showing the effect of withdrawal of each individual factor from previously described UB culturing condition[1] as indicated. C1, CHIR99021 at the concentration of 1 μM. Scale bars, 200 μm. **d**, Bright field images showing the improved UB branching by increasing CHIR99021 concentration from 1μM (C1) to 3μM (C3). Scale bars, 200 μm. **e**, Bright field (BF) and fluorescence (*Wnt11*-RFP) images showing improved *Wnt11*-RFP expression by replacing FGF1 with FGF9. Scale bars, 200 μm. **f**, **g**, Summary of screening results of the 1st round screening in Stage III for identifying individual hits. In the columns of "*Wnt11*-RFP" and "Organoid growth": "-" indicates no significant differences were observed compared to the control group (FGF9+C3+TT+GDNF). In the column of "Selected for R2 screening": "Y" indicates the factor was selected for 2nd round (R2) screening; "-" indicates the factor was not selected. **h-j**, Bright field (BF) and fluorescence (*Wnt11*-RFP) images showing improved *Wnt11*-RFP expression in UB organoids with the addition of LDN (h), A83 (i), and Rspo1 (j), as compared to their corresponding controls, in the 1st round screening in Stage III. LDN, LDN193189; A83, A83-01; Rspo1, R-Spondin 1. Scale bars, 200 μm. **k**, Bright field (BF) and fluorescence (*Wnt11*-RFP) images showing improved *Wnt11*-RFP expression in UB organoid with the addition of both JAK Inhibitor I and SB202190 (+JK/SB), as compared to the corresponding control (-JK/SB), in the 2nd round screening in Stage III. Scale bars, 200 μm. **l**, Bright field (BF) and fluorescence (*Wnt11*-RFP) images showing the morphology and *Wnt11*-RFP reporter expression in the UB organoids cultured in complete UBCM or upon withdrawal of indicated components from the UBCM for 9 days. TT, TTNPB. Scale bars, 200 μm.

Zeng *et al*., Supplementary Figure 2

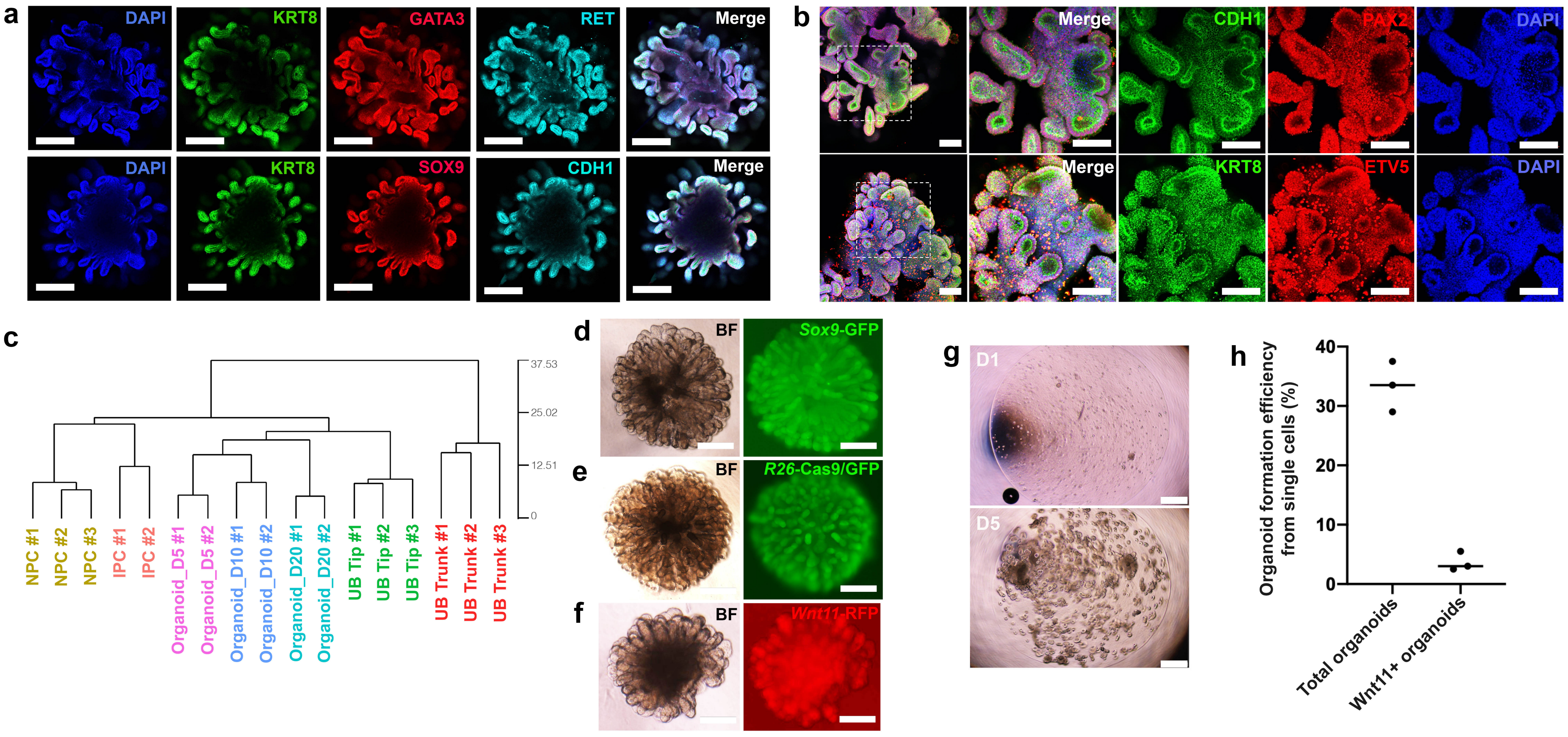

Supplementary Figure 2. Derivation and characterization of mouse UB organoid. a, **b**, Immunostaining of the branching UB organoid at day 10 of culture for various UB markers. Note the red signals that do not overlay with DAPI from PAX2 and ETV5 staining panels are non-specific signals. In **b**, The four panels on the right represent the boxed region in the left panel. Scale bars, a, 200 µm; b, 100 µm. **c**, Unsupervised clustering analysis of RNA-seq data. **d**, Bright field (BF) and fluorescence images of a UB organoid derived from *Sox9*-GFP genetic background. Scale bars, 200 µm. **e**, Bright field (BF) and fluorescence images of a UB organoid derived from *Rosa26*-Cas9/GFP (*R26*-Cas9/GFP) genetic background. Scale bars, 200 µm. **f**, Bright field (BF) and fluorescence images of a *Wnt11*-RFP UB organoid revived from freezing. Scale bars, 200 µm. **g**, Bright field images showing 200 single cells embedded into a drop of Matrigel and cultured in mUBCM for 1 day (D1) and 5 days (D5). Scale bars, 500 µm. **h**, Efficiency of UB organoid formation from 200 single UB cells. Each group represents 3 biological replicates. All data are presented as mean ± s.d. Source data are provided as a Source Data file.

Zeng *et al.*, Supplementary Figure 3

a

Culture Conditions	Base Medium	FGF9	EGF	Y27632
R1-1	hBI	+	-	-
R1-2	hBI	+	-	+
R1-3	hBI	+	+	-
R1-4	hBI	+	+	+
R1-5	APEL	+	-	-
R1-6	APEL	+	-	+
R1-7	APEL	+	+	-
R1-8	APEL	+	+	+

b

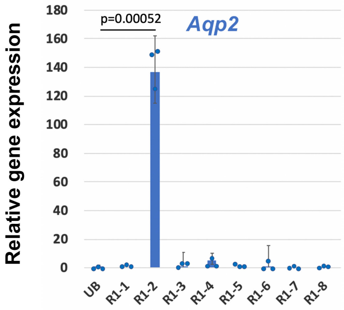

c

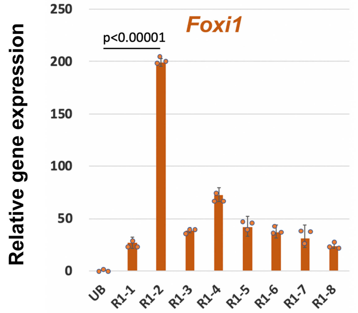

d

Culture Conditions	Base Condition	Chemical Tested	Selected for R3 Screen	Note
R2-1	R1-2	Control	-	
R2-2	R1-2	+ activin A	-	Dramatic cell death
R2-3	R1-2	+ A83-01	-	
R2-4	R1-2	+ BMP4	-	
R2-5	R1-2	+ BMP7	Y	
R2-6	R1-2	+ LDN193189	-	
R2-7	R1-2	+ Purmorphamine	-	
R2-8	R1-2	+ KAAD-Cyclopamine	-	
R2-9	R1-2	+ JAG-1	Y	
R2-10	R1-2	+ DAPT	Y	
R2-11	R1-2	+ CHIR99021	-	
R2-12	R1-2	+ IWR-1	-	
R2-13	R1-2	+ TTNPB	-	
R2-14	R1-2	+ LE135	-	
R2-15	R1-2	+ LIF	-	
R2-16	R1-2	+ JAKI	Y	
R2-17	R1-2	+ FGF1	-	
R2-18	R1-2	+ FGF2	-	
R2-19	R1-2	+ FGF7	-	
R2-20	R1-2	+ FGF10	-	
R2-21	R1-2	+ SP600126	-	Dramatic cell death
R2-22	R1-2	+ SB202190	-	
R2-23	R1-2	+ PD0325901	Y	
R2-24	R1-2	+ Aldosterone	Y	
R2-25	R1-2	+ Vasopressin	Y	

e

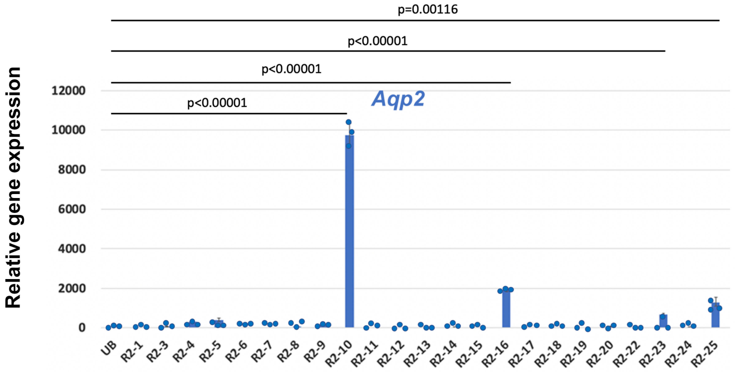

f

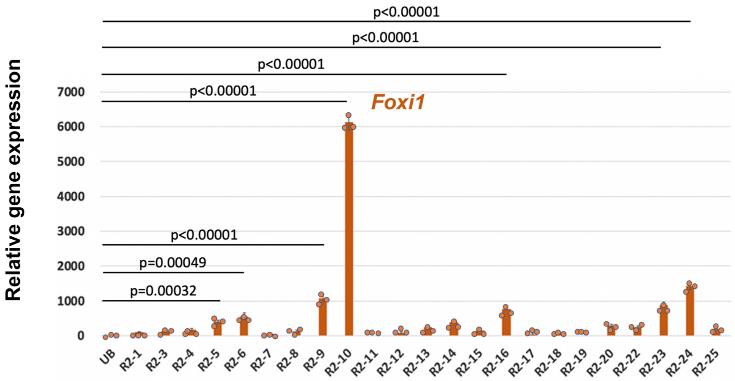

g

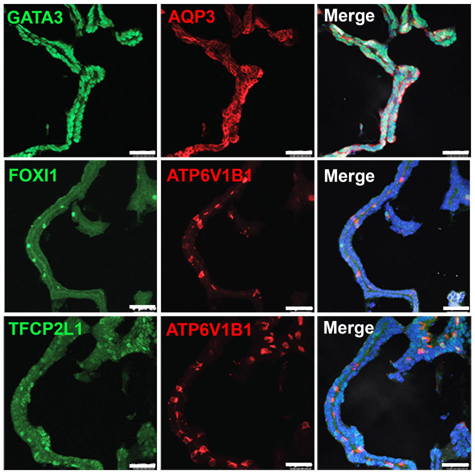

h

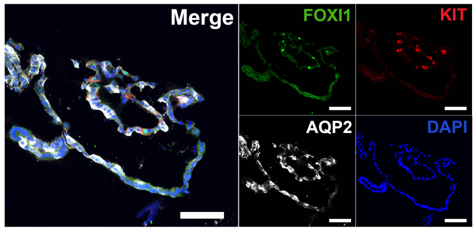

k **l** **m** **n**

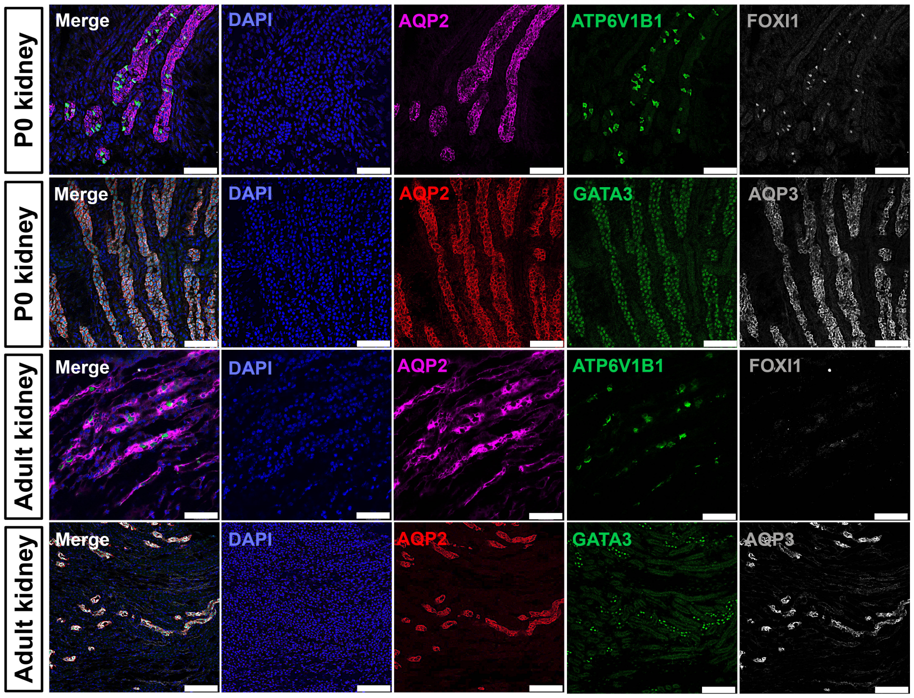

i

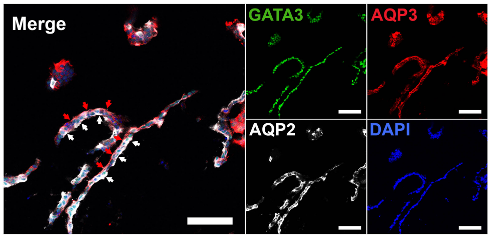

j

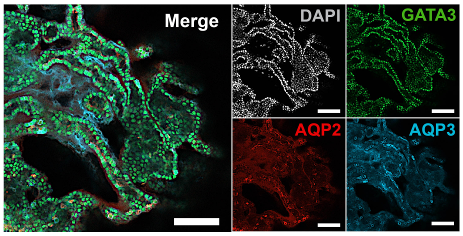

o

Genetic background of the UB organoids	*Wnt11-RFP*	*Wnt11-RFP*	*Rosa26-Cas9/GFP*
Starting Materials	Intact T-shaped UB	Intact T-shaped UB	Intact T-shaped UB
Passage Method	Manual	Single Cell	Single Cell
# of CD induction Tested	97	71	27
# of Successful CD induction	97	71	27
Differentiation Competency	100%	100%	100%

Supplementary Figure 3. Expandable UB organoid-based screening for CD differentiation. **a**, Summary of conditions tested in the 1st round of CD differentiation condition screening. **b**, **c**, qRT-PCR analyses of the 1st round of CD differentiation condition screening for PC marker gene *Aqp2* (b, in blue) and IC marker gene *Foxi1* (c, in orange). **d**, Summary of chemicals tested in the 2nd round of CD differentiation condition screening with the R1-2 medium as base medium. **e**, **f**, qRT-PCR analysis of the 2nd round of CD differentiation condition screening for PC marker gene *Aqp2* (e, in blue) and IC marker gene *Foxi1* (f, in orange). Note that data for R2-2 and R2-21 are not presented here, because dramatic cell death was observed in those conditions and were thus excluded from this analysis. **g-j**, Immunostaining of cryo-section (g-i) and whole-mount (j) samples of differentiated CD organoids for ureteric lineage marker (GATA3) and various PC (AQP2 and AQP3) and IC markers (FOXI1, ATP6V1B1, and KIT), and TFCP2L1, which is expressed stronger in the IC than PC at the protein level[2]. Note the sporadic distribution of the IC in the organoid. Scale bars, 50 µm. **k-n**, Immunostaining of cryo-section samples of postnatal day 0 kidney (P0, k and l) and adult mouse kidney (m and n) CD cells for ureteric lineage marker (GATA3) and various PC (AQP2 and AQP3) and IC markers (FOXI1 and ATP6V1B1). Note the sporadic distribution of the IC in the organoid. Scale bars, k and l, 50 µm; m and n, 100 µm. **o**, Summary of CD organoid derivation efficiency. All data are presented as mean ± s.d. Source data are provided as a Source Data file.

Zeng *et al.*, Supplementary Figure 4

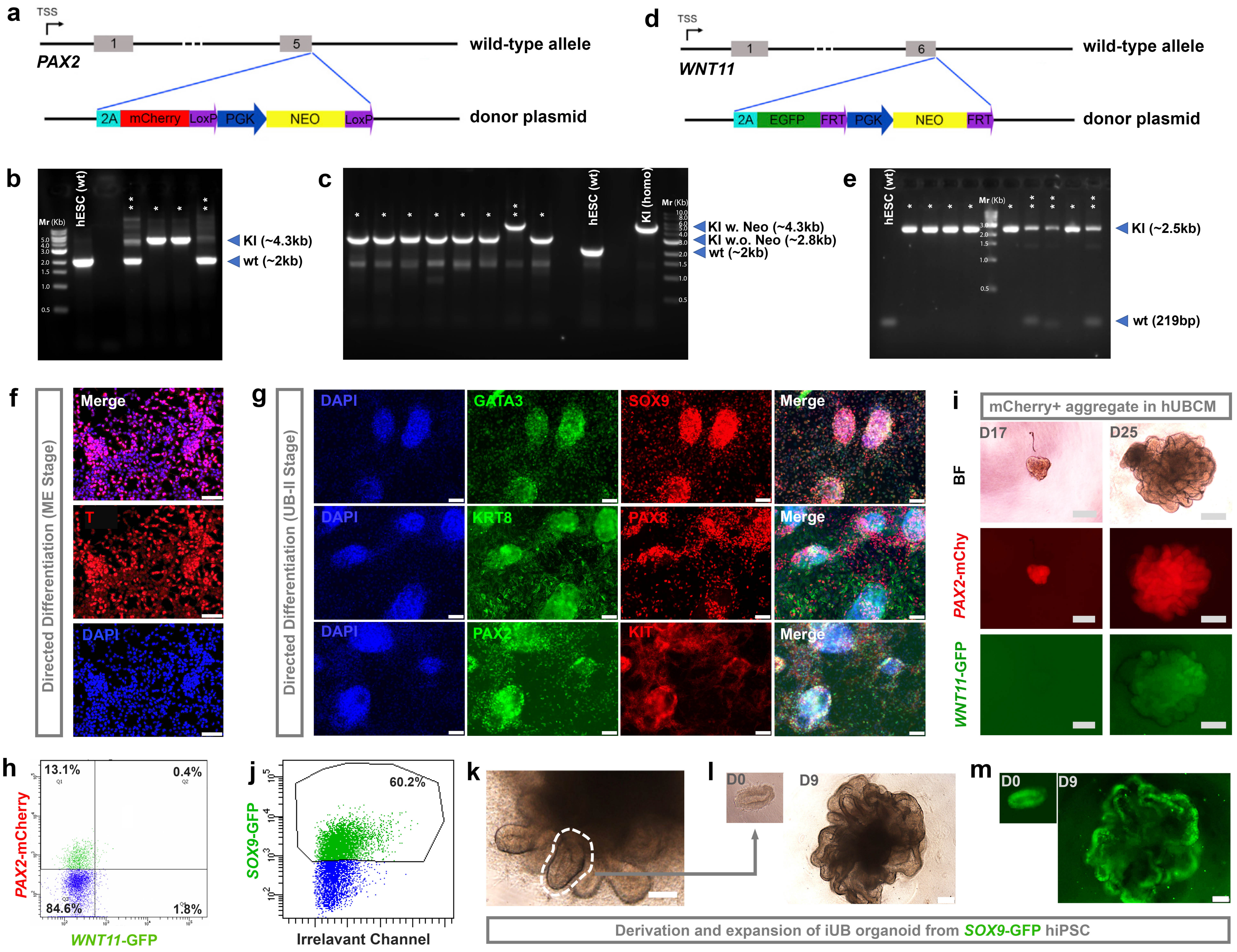

Supplementary Figure 4. Genetic engineering hESC with a dual reporter system by CRISPR/Cas9 and generation of iUB organoids from various hPSC reporter lines. a, Schematic of the genetic engineering of *PAX2*-mCherry reporter into the hESC line. See Supplementary Methods for more details. **b**, PCR-based genotyping results of *PAX2*-mCherry reporter knockin. Wild-type (wt) PCR product is 2011 bp, knockin (KI) product is 4308 bp. *, biallelic KI clones; **, monoallelic KI clones; wild-type hESC “hESC (wt)” is used as a non-editing control. **c**, PCR-based genotyping result for Cre-based excision of PGK-Neo cassette. PCR product for *PAX2*-mCherry knockin with PGK-Neo excised (KI w.o. Neo) is 2779 bp. *, clones with biallelic excision of PGK-Neo; **, clones without PGK-Neo excision; wild-type hESC “hESC (wt)”, and biallelic KI parental clone “KI (homo)” is used as controls. **d**, Schematic of the engineering of *WNT11*-GFP reporter into the *PAX2*-mCherry reporter hESC line. See Supplementary Methods for more details. **e**, PCR-based genotyping result for *WNT11*-GFP reporter knockin. Wild-type (wt) PCR product is 219 bp, knockin (KI) product is 2528 bp. *, biallelic KI clones; **, monoallelic KI clones; wild-type hESC “hESC (wt)” is used as a non-editing control. **f**, Immunostaining of hESC-derived mesendoderm cells (at the end of ME stage) for mesendoderm marker T (Brachyury). Scale bars, 100 µm. **g**, Immunostaining of hESC-derived UB precursor cells (at the end of UB-II stage and prior to FACS sorting) for various UB markers. Scale bars, 100 µm. **h**, Flow cytometry analysis of $mCherry^+$ and GFP^+ cells differentiated from *WNT11*-GFP/*PAX2*-mCherry dual reporter hESCs. **i**, Bright field (BF) and fluorescence images showing the induction of *WNT11*-GFP expression in the $mCherry^+$ aggregate upon extended culture in hUBCM. Scale bars, 200 µm. **j**, Flow cytometry analysis of GFP+ cells differentiated from *SOX9*-GFP reporter hiPSCs. **k-m**, Bright field (k and l) and *SOX9*-GFP (m) fluorescence images of branching iUB organoid derived from *SOX9*-GFP reporter hiPSCs in a typical passage cycle at day 0 (D0) and day 9 (D9). The indicated budding structure shown in (k) was dissected and re-embedded into Matrigel for iUB expansion shown in (l and m). Scale bars, 200 µm.

Zeng *et al.*, Supplementary Figure 5

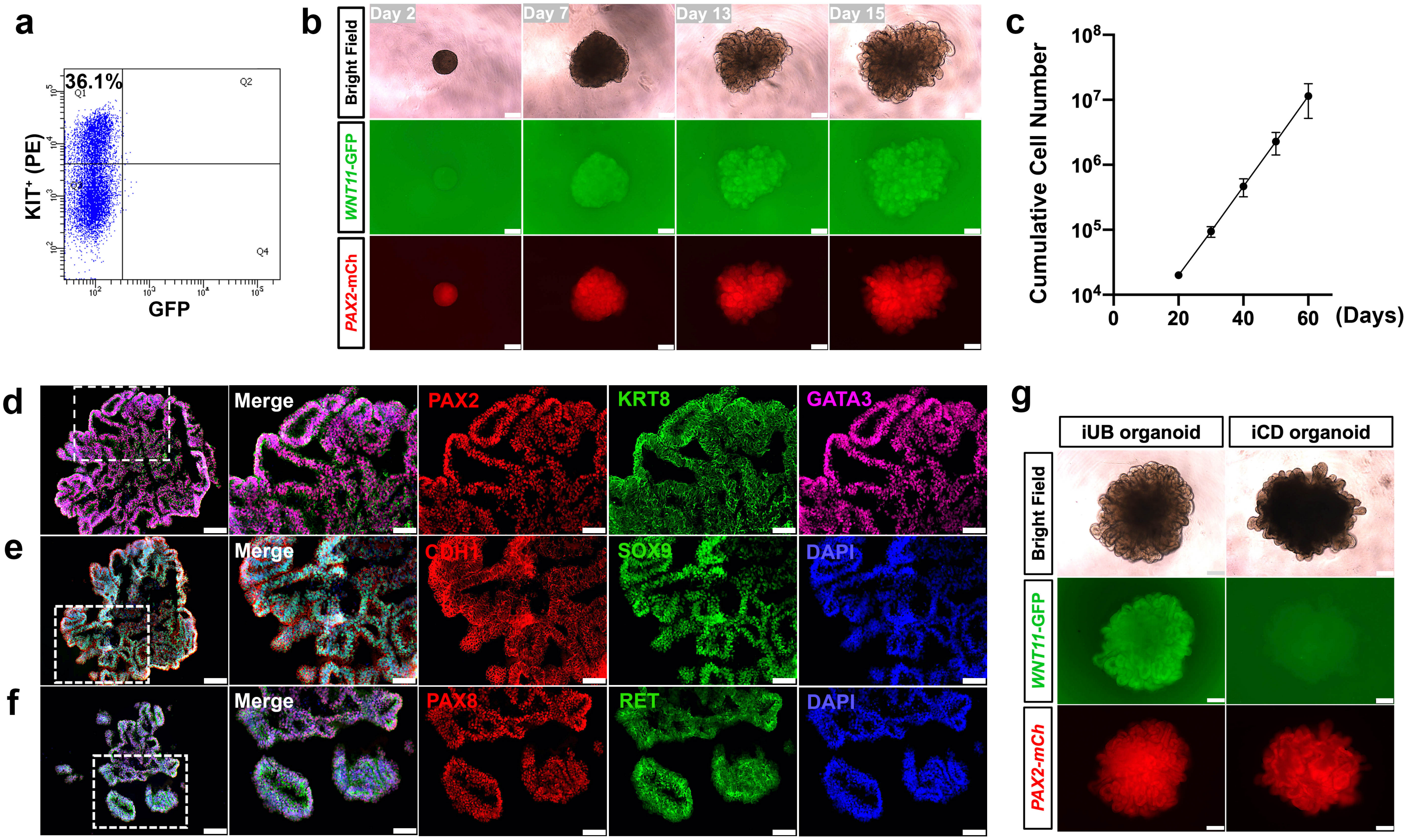

h

Origins of Human UB organoid	hESC	hESC	hiPSC
Cell lines	H1 (WA01)	*PAX2*-mCherry; *WNT11*-GFP	*SOX9*-GFP
Isolation Method	FACS (KIT+)	FACS (KIT+)	FACS (KIT+)
Passage Method	Manual or Single Cell	Manual or Single Cell	Manual or Single Cell
Freeze-Thaw	Yes	Yes	Yes
Typical Passage Cycle (days)	6-10	6-10	6-10
Passage #	8	8	8
In Vitro Cultured Time until submission (Days)	70	72	72
Reporter Expression	N/A	Yes	Yes
Fold of UPC expansion	10^8~10^9	10^8~10^9	10^7~10^8

Supplementary Figure 5. Generating human iUB and iCD organoids from the dual-reporter hPSC line independent of its reporters. **a**, Flow cytometry analysis of KIT^+ precursor cells differentiated from the dual reporter hESC line. **b**, Bright field (BF) and fluorescence images showing the induction of *WNT11*-GFP expression in the KIT^+ aggregate upon continued culture in hUBCM-v2. Scale bars, 200 μm. **c**, Cumulative growth curve of iUB organoid culture starting from 20,000 cells at day 20. Each time point represents 3 biological replicates. **d-f**, Immunostaining of cryo-section samples of the expandable iUB organoid for various UB markers. The four panels on the right represent the boxed region in the left panel. Scale bars, left panel, 100 μm; right 4 panels, 50 μm. **g**, Bright field and fluorescence images showing the morphological changes and decreasing of *WNT11*-GFP from iUB organoid (left panels) to mature iCD organoid (right panels). Scale bars, 200 μm. **h**, Summary of human iUB organoids derivation from different hPSC lines independent of reporter and their expansion *in vitro*. Total culture time are up until manuscript submission. The maximum organoid culture time and expansion could be longer. All data are presented as mean ± s.d. Source data are provided as a Source Data file.

Zeng *et al.*, Supplementary Figure 6

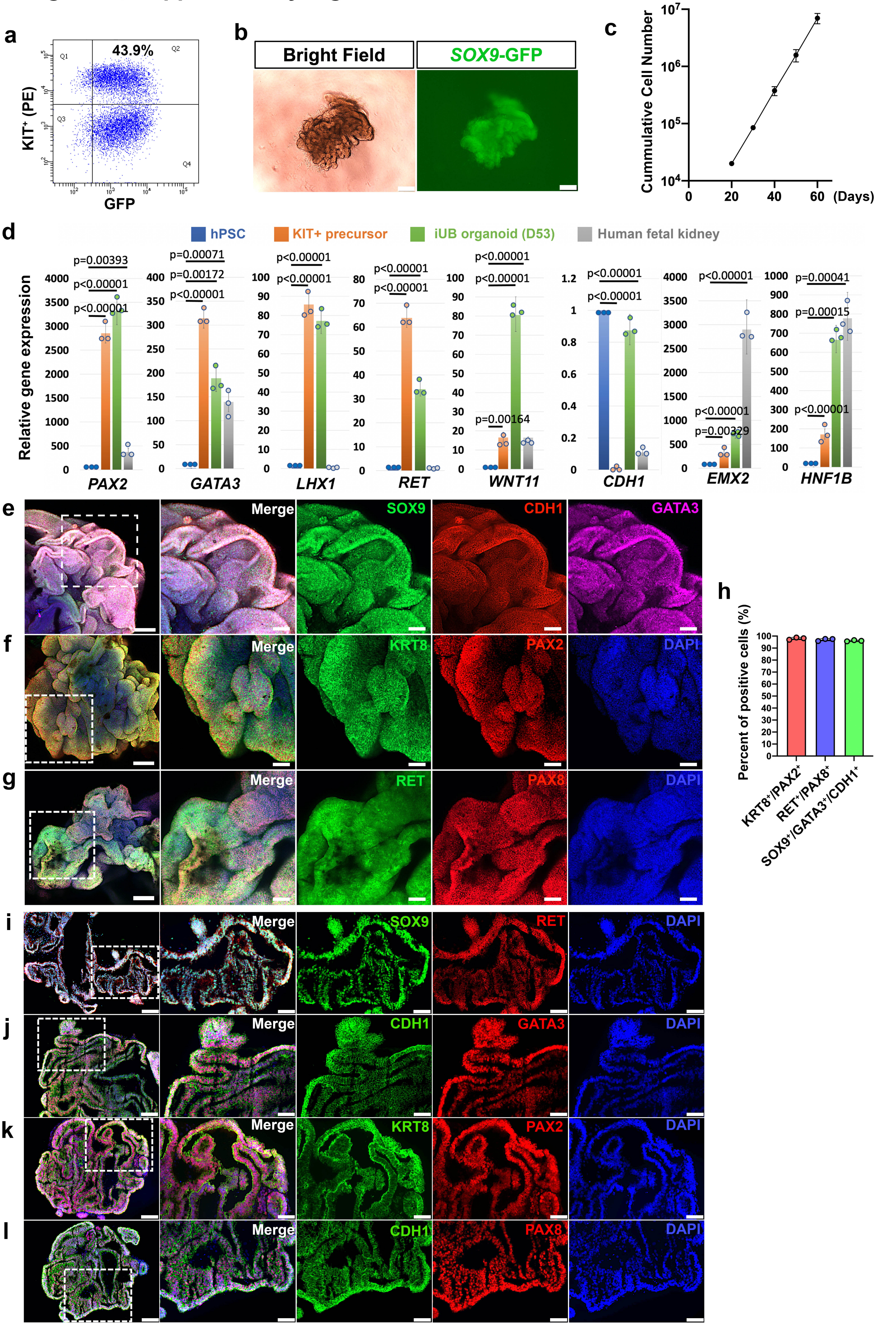

Supplementary Figure 6. Generating human iUB organoids from the *SOX9*-GFP iPSC independent of its reporter. a, Flow cytometry analysis of KIT^+ precursor cells differentiated from the *SOX9*-GFP hiPSC line. Note the *SOX9*-GFP reporter expression is induced in the majority of the cells using this new differentiation protocol. **b**, Bright field (BF) and fluorescence images showing the *SOX9*-GFP expression in the KIT^+ iUB organoid cultured in hUBCM-v2. Scale bars, 200 µm. **c**, Cumulative growth curve of iUB organoid culture starting from 20,000 cells at day 20. Each time point represents 3 biological replicates. **d**, qRT-PCR analyses of the FACS purified KIT^+ precursor (orange) and KIT^+ iUB organoids cultured for 53 days (D53, green) for various UB markers. Undifferentiated hiPSCs (dark blue) and human fetal kidney (gray, 11.2 week gestational age) were used as controls. The significance was determined by two-tailed unpaired Student's t-tests; n=3. **e-g** and **i-l**, Immunostaining of whole-mount (e-g) and cryo-section (i-l) samples of the expandable iUB organoid for various UB markers. The four panels on the right represent the boxed region in the left panel. Scale bars, e-g, left panels, 100 µm; right 4 panels, 40 µm; i-l, left panels, 100 µm; right 4 panels, 50 µm. **h**, Quantification of percentages of iUB cells stained positive for different UB markers in Supplementary Fig. 6e-g. Each column represents counts from 3 different fields of view (n=3). All data are presented as mean ± s.d. Source data are provided as a Source Data file.

Zeng *et al.*, Supplementary Figure 7

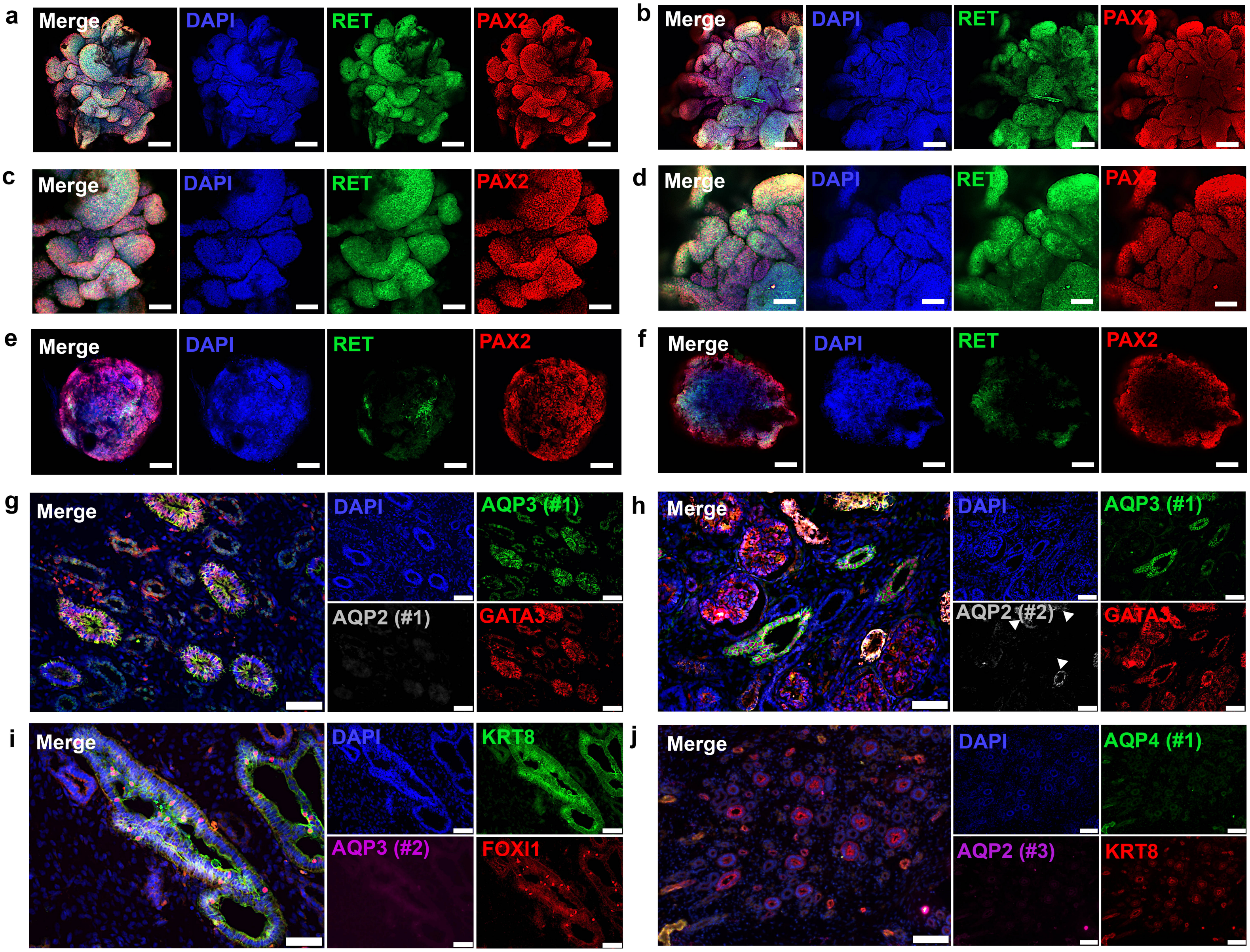

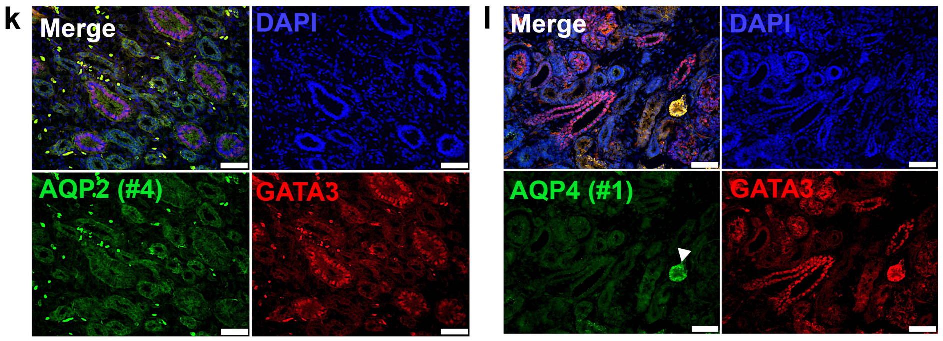

m

Summary of PC/IC antibody test result in 17.5-week human fetal kidney sections				
PC/IC Antibodies Tested	Company	Catalog #	Dilution	Test Result
AQP2 #1 (Fig. S7g)	Santa Cruz	sc-515770	1:100	X
AQP2 #2 (Fig. S7h)	Santa Cruz	sc-47710	1:100	X
AQP2 #3 (Fig. S7j)	Santa Cruz	sc-9882	1:100	X
AQP2 #4 (Fig. S7k)	Abcam	ab62628	1:100	X
AQP3 #1 (Fig. S7g and h)	Abcam	ab125219	1:100	✓
AQP3 #2 (Fig. S7i)	Santa Cruz	sc-518001	1:100	X
AQP4 #1 (Fig. S7j and l)	Santa Cruz	sc-32739	1:100	X
FOXI1 (Fig. S7i)	Novus Biologicals	NB300-926	1:300	✓

Supplementary Figure 7. Immunostaining of whole-mount samples of control and RET KO iUB organoids and human CD antibodies testing. a-f, Corresponding to Fig. 6g. Whole-mount immunostaining of the control (**a-d**) and *RET* KO (**e** and **f**) human iUB organoids for various UB markers. **a**, **c**, and **e** were from the experimental group with sgRNA #1; **b**, **d**, and **f** were from the experimental group with sgRNA #2. Scale bars, a and b, 100 μm; c-f, 40 μm. **g-l**, Immunostaining of 17.5 week gestational age human fetal kidney cryo-section samples for testing various human CD marker antibodies, including PC markers AQP2, AQP3, AQP4, and IC markers FOXI1. Arrow heads in (h) and (l) indicate non-specific signals. Scale bars, g-i, k, l, 50 μm; j, 100 μm. **m**, Summary of all CD antibodies we have tested in human kidney samples.

Zeng *et al.*, Supplementary Figure 8

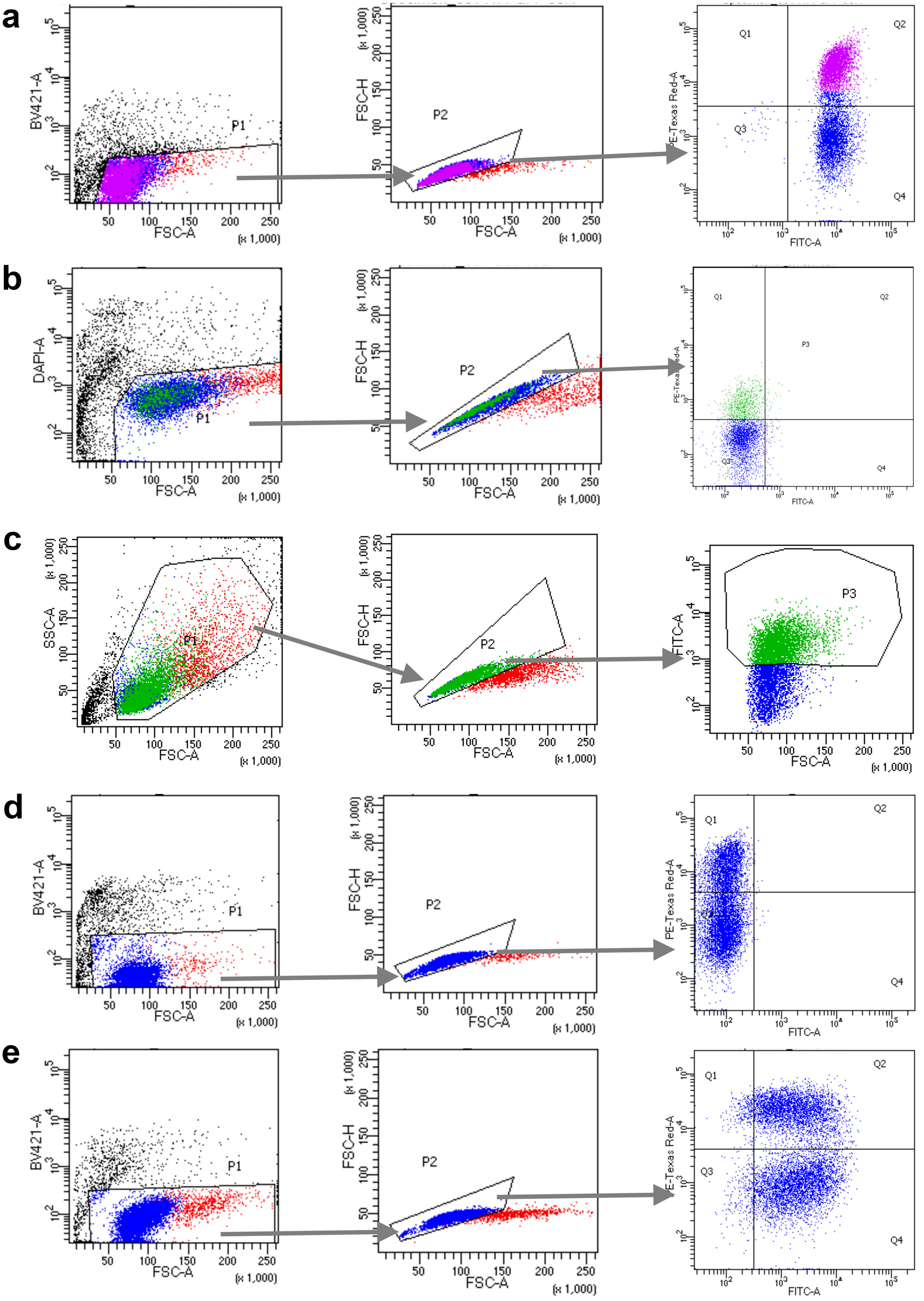

Supplementary Figure 8. FACS sequential gating/sorting strategies used in iUB differentiation. a, Corresponding to Fig. 5i, gating strategy for detection of KIT^+ precursor cells differentiated from wild-type H1 hESC. **b,** Corresponding to Supplementary Fig. 4h, gating strategy for detection of $mCherry^+$ and GFP^+ cells differentiated from *WNT11*-GFP/*PAX2*-mCherry dual reporter hESCs. **c,** Corresponding to Supplementary Fig. 4j, gating strategy for detection of GFP+ cells differentiated from *SOX9*-GFP reporter hiPSCs. **d,** Corresponding to Supplementary Fig. 5a, gating strategy for detection of KIT^+ precursor cells differentiated from the dual reporter hESC line. **e,** Corresponding to Supplementary Fig. 6a, gating strategy for detection of KIT^+ precursor cells differentiated from the *SOX9*-GFP hiPSC line**.**

Supplementary Table 1. Summary of available in vitro models for mouse kidney branching morphogenesis

		This study	Yuri *et al.*, 2017[1]	Taguchi et al., 2017[3]
UB Stage	Source of UB	E11.5 UB	E11.5 UB	mESC
	UB branching period in vitro	Up to 3 weeks	Up to 9 days	6-7 days
	Identity of branching UB	UPC	Mixture of UB tip and trunk	Not shown
	Repetitive passaging for expansion	Yes	Not shown	Not shown
	Freeze & thaw	Yes	Not shown	Not shown
	UB expansion fold	10^5-10^6 fold	Not shown	Not shown
	Gene manipulation (overexpression)	Yes	Not shown	Not shown
	Gene manipulation (knockout by Cas9)	Yes	Not shown	Not shown
CD Stage	UB-to-CD differentiation method	Yes	Not shown	
	PC & IC generation	Yes		
	Proper 3D patterning of PC & IC	Yes		

Supplementary Table 2. Summary of available in vitro models for human kidney branching morphogenesis

			This study	Howden et al., 2020[4]	Uchimura et al., 2020[5]	Mae et al., 2020[6]	Tsujimoto *et al.*, 2020[7]	Mae *et al.*, 2018[8]	Taguchi *et al.*, 2017[3]	Xia *et al.*, 2013[9]
**Starting from primary human UB**	**UB Stage**	**UPC purification**	FACS for *RET*$^+$	Not shown						
		**UPC expansion period**	>100 days							
		**UPC expansion fold**	$>10^{10}$							
	**CD Stage**	**UB-to-CD differentiation**	Yes							
		**PC & IC generation**	Yes							
**Starting from human PSC**	**UB Stage**	**Generation of UB-like cells**	Yes	Yes	Yes	Yes	Yes	Yes	Yes	Yes
		**Identity of the UB-like cells generated**	UPC	UPC	Anterior intermediate mesoderm	Mixture of tip and trunk cells	Mixture of tip and trunk cells	Mixture of tip and trunk cells	Mixture of tip and trunk cells	Precursor of UB
		**Time needed to generate pure UPC**	12 days	>34 days	N/A					
		**UPC expansion period**	>70 days	Up to 3 weeks shown						
		**UPC expansion fold**	$>10^9$	Not shown						
	**CD Stage**	**Directed UB-to-CD differentiation**	Yes	Yes	No (co-culture with posterior intermediate mesoderm)	Yes	No (co-culture with nephron progenitor cells)	Not shown		
		**PC & IC generation**	Yes (evidence from qRT-PCR and IF)	Yes (evidence from RNA-seq)	Yes (evidence from qRT-PCR, IF and RNA-seq)	No ("CD progenitor" generated)	Yes (evidence from IF)			

Supplementary table 3. Key resources.

Antibodies

Antibodies	SOURCE	IDENTIFIER	DILUTION
KRT8	DSHB	Cat.# TROMA-I	1:50
GATA3	R&D Systems	Cat.# AF2605	1:400
SOX9	Abcam	Cat.# 185230	1:400
RET (mouse)	Cell Signaling Technology	Cat.# 3223S	1:300
RET (human)	R&D Systems	Cat.# AF1485	1:100
PAX2	Covance	Cat.# PRB-276P	1:400
PAX8	Proteintech	Cat.# 10336-1-AP	1:400
ETV5	Abcam	Cat.# Ab102010	1:300
CDH1	BD	Cat.# 610181	1:400
AQP2	Santa Cruz	Cat.# sc-47710	1:300
AQP3	Abcam	Cat.# ab125219	1:100
FOXI1	Novus Biologicals	Cat.# NB300-926	1:300
ATP6V1B1	Abcam	Cat.# ab192612	1:300
TFCP2L1	R&D Systems	Cat.# AF5726	1:300
KIT	Cell Signaling	Cat.# 3074	1:300
PE-CD117(c-kit)	Biolegend	Cat.# 313204	1:200
SIX2	Proteintech	Cat.# 11562-1-AP	1:1000
PODXL(mouse)	R&D Systems	Cat.# MAB1556	1:500
WT1	Abcam	Cat.# ab89901	1:1000
LTL	Vector laboratories	Cat.# B-1325	1:500
T	R&D Systems	Cat.# AF2085	1:300

Chemicals, Peptides, and Recombinant Proteins

Reagent Name	SOURCE	IDENTIFIER	CONCENTRATIONS
GlutaMAX-I (100X)	Invitrogen	Cat.# 35050-079	1X
MEM NEAA (100X)	Invitrogen	Cat.# 11140-050	1X
2-Mercaptoethanol	Invitrogen	Cat.# 21985-023	0.1mM
Pen Strep (100X)	Invitrogen	Cat.# 15140-122	1X
B-27, minus vitamin A	Invitrogen	Cat.# 12587-010	1X
ITS (100X)	Sigma	Cat.# I3146-5ML	1X
FBS	Gemini	100-106	10%
DMEM	Invitrogen	11995-040	1x
DMEM/F12	Invitrogen	11330-032	1x
APEL2	STEMCELL Technologies	Cat.# 05270	1x
LDN-193189	Reagents Direct	Cat.# 36-F52	30nM, 200nM
TTNPB	TOCRIS	Cat.# 0761	0.1μM
CHIR99021	Reagents Direct	Cat.# 27-H76	3μM
JAK Inhibitor I	Stemcell Technologies	Cat.# 74022	100nM
GDNF (mouse)	PeproTech	Cat.# 450-44-50μg	50ng/ml
GDNF (human)	PeproTech	Cat.# 450-10-50μg	50ng/ml
A83-01	STEMGENT	Cat.# 04-0014	0.2μM
R-Spondin 1	R&D Systems	Cat.# 4645-RS-100	100ng/ml
SB202190	Axon Medchem	Cat.# Axon 1304	5μM
Y27632	Cayman Chemical	Cat.# 10005583	10μM
FGF9	R&D Systems	Cat.# 273-F9-025	50ng/ml
FGF2	Stemcell Technologies	Cat.# 78003	50,100, 200ng/ml
FGF7	PeproTech	Cat.# 100-19	50ng/ml

FGF1	Stemcell Technologies	Cat.# 78187	50ng/ml
FGF10	PeproTech	Cat.# 100-26	50ng/ml
Aldosterone	Sigma-Aldrich	Cat.# A9477	100nM
Vasopressin	Sigma-Aldrich	Cat.# V0377-100IU	1 I.U./ml
PD0325901	Reagents Direct	Cat.# 39-C68	1μM
DAPT	Sigma-Aldrich	Cat.# D5942	5μM
Activin A	R&D Systems	Cat.# 338-AC-010	10ng/ml, 50ng/ml
EGF	R&D Systems	Cat.# 236-EG-200	50ng/ml
BMP4	R&D Systems	Cat.# 314-BP-010	10ng/ml
BMP7	R&D Systems	Cat.# 354-BP-010	10ng/ml
Purmorphamine	STEMGENT	Cat.# 04-0009	1μM
KAAD-Cyclopamin	STEMGENT	Cat.# 04-0028	50nM
JAG-1	ANA SPEC	Cat.# AS-61298	1μM
IWR-1	Sigma	Cat.# 10161-5MG	2.5μM
LE-135	Tocris	Cat.# 2021	0.5μM
LIF	Millipore	Cat.# ESG1107	1000 units/ml
SP600126	Tocris	Cat.# 1496	10μM
FGF4	Peprotech	Cat.# 100-31	50ng/ml
FGF8	Peprotech	Cat.# AF-100-25	100ng/ml
FGF20	Peprotech	Cat.# 100-41	50ng/ml
VEGF	R&D Systems	Cat.# 293-VE-010	50ng/ml
Heparin	Sigma	Cat.# H3149-100KU	1μg/ml
SCF	R&D Systems	Cat.# 255-SC-010	50ng/ml
KSR	Thermo Fisher	Cat.# 10828028	3%, 15% (v/v)
LY294002	Selleck Chemicals	Cat.# S1105	5μM
Cyclosporine A	Cayman Chemical	Cat.# 12088	10μM
TNF-α	R&D Systems	Cat.# 210-TA-020	100ng/ml
HGF	Peprotech	Cat.# 315-23	50ng/ml
Foskolin	Sigma	Cat.# F3917-10MG	10μM
IGF1	Sigma	Cat.# I1271-.1MG	20ng/ml
IGF2	Peprotech	Cat.# AF-100-12	2ng/ml
AICAR	Tocris	Cat.# 2840	0.5mM
Metformin	Tocris	Cat.# 2864	1mM
Abbott	Tocris	Cat.# 1506	0.1mM
XMU-MP-1	Selleck Chemicals	Cat.# S8334	1μM
Verteporfin	Tocris	Cat.# 5305	1μM
PDGF-BB	R&D Systems	Cat.# 220-BB-010	10ng/ml
CloneR	STEMCELL Technologies	Cat. # 05888	1x
Matrigel	R&D Systems	Cat. # 3433-010-01	1x

Biological Samples

REAGENT	SOURCE	IDENTIFIER
Human fetal kidney tissues (9-13 week)	USC and CHLA	N/A

Critical Commercial Assays

REAGENT	SOURCE	IDENTIFIER
TRIzol Reagent	Thermo Fisher Scientific	Cat.# 15596026
Direct-zol RNA MicroPrep Kit	Zymo Research	Cat.# R2062
iScript Reverse Transcription Supermix	Bio-Rad	Cat.# 1708841
PowerUp SYBR Green Master Mix	Thermo Fisher Scientific	Cat.# A25777
KAPA Stranded mRNA-Seq Kit	KAPA Biosystems	Cat.# KK8420

Deposited Data

RESOURCE	SOURCE	IDENTIFIER
RNA-seq for cultured UB organoid	This study	NCBI GEO: GSE149109

Experimental Models: Cell lines

RESOURCE	SOURCE	IDENTIFIER
Mouse UB organoid (*Wnt11*-RFP)	This study	N/A
Mouse UB organoid (*Sox9*-GFP)	This study	N/A
Mouse UB organoid (*Hoxb7*-Venus)	This study	N/A
Mouse UB organoid (Swiss Webster)	This study	N/A
Human UB organoid (from fetal kidney)	This study	N/A
Human UB organoid (from hESC)	This study	N/A
Human UB organoid (from hiPSC)	This study	N/A
Human ESC: *Wnt11*-GFP/*Pax2*-mCherry H1 line	This study	N/A
Human ESC: Wild-type H1 line	University of Wisconsin-Madison, WiCell	hPSCReg ID: WAe001-A
Human iPSC: *Sox9*-GFP line	University of Texas Health Science Center at Houston	N/A
3D cultured NPC (wild-type)	Zhongwei Li Lab, USC	N/A

Experimental Models: Organisms/Strains

RESOURCE	SOURECE	IDENTIFIER
Swiss Webster	Taconic Biosciences	Model # SW-F, MPF 4 weeks
Wnt11-RFP	The Jackson Laboratory	JAX # 018683
Sox9-GFP	Gifu University, Japan	N/A
Hoxb7-Venus	The Jackson Laboratory	JAX # 016252
Rosa26-Cas9/GFP	The Jackson Laboratory	JAX #026179

Oligonucleotides

RESOURCE	SOURECE	IDENTIFIER
Genotyping primers for dual reporter knockin	This study	Supplementary Table 4
qRT-PCR primers	This study	Supplementary Table 5

Supplementary table 4. Genotyping primer sequences for detecting *PAX2*-mCherry and *WNT11*-GFP knockin (KI).

PCR Primers		
**Reporter Name**	**Forward Primer**	**Reverse Primer**
***PAX2*-mCherry KI**	TCCCATTTCCACCCATTAGGGGCCA	GCATCAAGTAACTGCTGGAGGAAGACC
***WNT11*-GFP KI**	ACAAGACATCCAACGGAAGC	TGAGGGTCCTTGAGCAGAGT

Supplementary table 5. qRT-PCR Primer sequences.

qRT-PCR Primers (Mouse)		
**Gene Name**	**Forward Primer**	**Reverse Primer**
Gapdh	CATGGCCTTCCGTGTTCCTA	CCTGCTTCACCACCTTCTTGAT
Wnt11	TGTGCGGACAACCTCAGCTAC	ATGGCATTTACACTTCGTTTCCAG
Ret	TGAGCCCTCGGCAACATTC	GCCTCTGATGACAGCAATACTGGA
Aqp2	GCCACCTCCTTGGGATCTAT	TGTAGAGGAGGGAACCGATG
Aqp3	GGCGCTGGGATTGTTTTTGG	GCCAGAGACGAAAAGCTCATT
Foxi1	AGTACGTGGCCGACAACTTTC	AGTCCAGTAATTCCCTTTGCCT
Tfcp2l1	GCTGGAGAATCGGAAGCTAGG	AAAACGACACGGATGATGCTC
Atp6v1b1	GGCTGTGACCCGAAACTACAT	CTCAGCATACTGGGCAAACTT
Slc4a1	CTAGTGGGCCGGGCTAATTTT	TGCGGAACACTCTTTCTGTCA
Slc26a4	CCTTTGGTGTGGTAAAGACTCTC	GACCGAACTGAACAGGTACTG

qRT-PCR Primers (Human)		
**Gene Name**	**Forward Primer**	**Reverse Primer**
GAPDH	GTGGACCTGACCTGCCGTCT	GGAGGAGTGGGTGTCGCTGT
PAX2	CCCAAAGTGGTGGACAAGAT	GAAAGGCTGCTGAACTTTGG
GATA3	CGTCCTGTGCGAACTGTCA	GTCCCCATTGGCATTCCTCC
LHX1	CTTCTTCCGGTGTTTCGGTA	TCATGCAGGTGAAGCAGTTC
RET	TATCCTGGGATTCCTCCTGA	TCTCCAGGTCTTTGCTGATG
WNT11	ATGTGCGGACAACCTCAGCTAC	GATGGAGCAGGAGCCAGACA
CDH1	ACTCGTAACGACGTTGCACCA	GGTCAGTATCAGCCGCTTTCAG
EMX2	AATGCGGCGAAGACTCTGG	TTTAGACGAGGGTCGCTTGTTG
HNF1B	GCTGTGACTCAGCTGCAGAACTC	TGTACTGATGCTGCTGGTATCTGTG
ETV5	CAGTCAACTTCAAGAGGCTTGG	TGCTCATGGCTACAAGACGAC
SOX9	AGCGAACGCACATCAAGAC	CTGTAGGCGATCTGTTGGGG
GFRA1	AAGCACAGCTACGGAATGCT	GTTGGGCTTCTCCCTCTCTT
AQP2	CTGGTACAGGCTCTGGGCCACATAA	ATGTCTGCTGGCGTGATCTCATGGAG
AQP3	AGACAGCCCCTTCAGGATTT	TCCCTTGCCCTGAATATCTG
AQP4	CATGGAAATCTTACCGCTGGT	TCAGTCCGTTTGGAATCACAG
FOXI1	AACTCACTGACCTTCAACTCCT	CCATAGCTGAGCATGTTGGT

Supplementary table 6. Medium recipe of mUBCM (mouse UB culture medium).

Supplements:

Reagent Name	Company	Cat. No.	Final Concentration
GlutaMAX-I (100X)	Invitrogen	35050-079	1X
MEM NEAA (100X)	Invitrogen	11140-050	1X
2-Mercaptoethanol	Invitrogen	21985-023	0.1mM
Pen Strep (100X)	Invitrogen	15140-122	1X
B-27, minus vitamin A	Invitrogen	12587-010	1X
ITS (100X)	Sigma	I3146-5ML	1X
LDN-193189	Reagents Direct	36-F52	200nM
TTNPB	TOCRIS	0761	0.1µM
CHIR99021	Reagents Direct	27-H76	3µM
JAK Inhibitor I	Stemcell Technologies	74022	100nM
GDNF (mouse)	PeproTech	450-44-50µg	50ng/ml
A83-01	STEMGENT	04-0014	0.2µM
Rspo1	R&D Systems	4645-RS-100	100ng/ml
FGF9	R&D Systems	273-F9-025	50ng/ml
SB202190	Axon Medchem	Axon 1304	5µM

Basal medium: DMEM/F12 (1:1) (1X), Invitrogen, Cat. No. 11330-032.

Supplementary table 7. Medium recipe of hUBCM (Human UB culture medium).

Supplements:

hUBCM-v1

Reagent Name	Company	Cat. No.	Final Concentration
GlutaMAX-I (100X)	Invitrogen	35050-079	1X
MEM NEAA (100X)	Invitrogen	11140-050	1X
2-Mercaptoethanol	Invitrogen	21985-023	0.1mM
Pen Strep (100X)	Invitrogen	15140-122	1X
B-27, minus vitamin A	Invitrogen	12587-010	1X
ITS (100×)	Sigma	I3146-5ML	1X
LDN-193189	Reagents Direct	36-F52	200nM
TTNPB	TOCRIS	0761	0.1μM
CHIR99021	Reagents Direct	27-H76	3μM
JAK inhibitor I	Stemcell Technologies	74022	100nM
GDNF (human)	PeproTech	450-10-50μg	50ng/ml
A83-01	STEMGENT	04-0014	0.2μM
Rspo1	R&D Systems	4645-RS-100	100ng/ml
FGF7	PeproTech	100-19	50ng/ml
SB202190	Axon Medchem	Axon 1304	5μM
Y27632	Cayman Chemical	10005583	10μM
EGF	R&D Systems	236-EG-200	50ng/ml

Basal medium: DMEM/F12 (1:1) (1X), Invitrogen, Cat. No. 11330-032.

hUBCM-v2

<table>
<tr><th>Reagent Name</th><th>Company</th><th>Cat. No.</th><th colspan="2">Final Concentration</th></tr>
<tr><td>GlutaMAX-I (100X)</td><td>Invitrogen</td><td>35050-079</td><td colspan="2">1X</td></tr>
<tr><td>MEM NEAA (100X)</td><td>Invitrogen</td><td>11140-050</td><td colspan="2">1X</td></tr>
<tr><td>2-Mercaptoethanol</td><td>Invitrogen</td><td>21985-023</td><td colspan="2">0.1mM</td></tr>
<tr><td>Pen Strep (100X)</td><td>Invitrogen</td><td>15140-122</td><td colspan="2">1X</td></tr>
<tr><td>B-27, minus vitamin A</td><td>Invitrogen</td><td>12587-010</td><td colspan="2">1X</td></tr>
<tr><td>ITS (100×)</td><td>Sigma</td><td>I3146-5ML</td><td colspan="2">1X</td></tr>
<tr><td>LDN-193189</td><td>Reagents Direct</td><td>36-F52</td><td colspan="2">200nM</td></tr>
<tr><td>TTNPB</td><td>TOCRIS</td><td>0761</td><td colspan="2">0.1μM</td></tr>
<tr><td rowspan="2">CHIR99021</td><td rowspan="2">Reagents Direct</td><td rowspan="2">27-H76</td><td>For iPSC-derived iUB</td><td>1 μM</td></tr>
<tr><td>All other hUB/iUB</td><td>3 μM</td></tr>
<tr><td>JAK inhibitor I</td><td>Stemcell Technologies</td><td>74022</td><td colspan="2">100nM</td></tr>
<tr><td>GDNF (human)</td><td>PeproTech</td><td>450-10-50μg</td><td colspan="2">50ng/ml</td></tr>
<tr><td>A83-01</td><td>STEMGENT</td><td>04-0014</td><td colspan="2">0.2μM</td></tr>
<tr><td>Rspo1</td><td>R&D Systems</td><td>4645-RS-100</td><td colspan="2">100ng/ml</td></tr>
<tr><td>FGF7</td><td>PeproTech</td><td>100-19</td><td colspan="2">50ng/ml</td></tr>
<tr><td>SB202190</td><td>Axon Medchem</td><td>Axon 1304</td><td colspan="2">5μM</td></tr>
<tr><td>EGF</td><td>R&D Systems</td><td>236-EG-200</td><td colspan="2">50ng/ml</td></tr>
</table>

Basal medium: DMEM/F12 (1:1) (1X), Invitrogen, Cat. No. 11330-032.

Supplementary table 8. Medium recipe of CDDM (CD differentiation medium).

Supplements:

mCDDM (mouse):

Reagent Name	Company	Cat. No.	Final Concentration
GlutaMAX-I (100X)	Invitrogen	35050-079	1X
MEM NEAA (100X)	Invitrogen	11140-050	1X
2-Mercaptoethanol	Invitrogen	21985-023	0.1mM
Pen Strep (100X)	Invitrogen	15140-122	1X
B-27, minus vitamin A	Invitrogen	12587-010	1X
ITS (100X)	Sigma	I3146-5ML	1X
FGF9	R&D Systems	273-F9-025	50ng/ml
Y27632	Cayman Chemical	10005583	10μM
Aldosterone	Sigma-Aldrich	A9477	100nM
Vasopressin	Sigma-Aldrich	V0377-100IU	1 I.U./ml
PD0325901	Reagents Direct	39-C68	1μM
DAPT	Sigma-Aldrich	D5942	5μM

Basal medium: DMEM/F12 (1:1) (1X), Invitrogen, Cat. No. 11330-032.

hCDDM (human):

Reagent Name	Company	Cat. No.	Final Concentration
GlutaMAX-I (100X)	Invitrogen	35050-079	1X
MEM NEAA (100X)	Invitrogen	11140-050	1X
2-Mercaptoethanol	Invitrogen	21985-023	0.1mM
Pen Strep (100X)	Invitrogen	15140-122	1X
B-27, minus vitamin A	Invitrogen	12587-010	1X
ITS (100X)	Sigma	I3146-5ML	1X
Aldosterone	Sigma-Aldrich	A9477	100nM
Vasopressin	Sigma-Aldrich	V0377-100IU	1 I.U./ml
KSR	Thermo Fisher	10828028	3% (v/v)

Basal medium: DMEM/F12 (1:1) (1X), Invitrogen, Cat. No. 11330-032.

Supplementary table 9. Medium recipe of stepwise directed differentiation to ND cells (D1 to D7 from hPSC).

Supplements:

Stages	Reagent Name	Final Concentration
Common supplements for all three stages	GlutaMAX-I (100X)	1X
	MEM NEAA (100X)	1X
	2-Mercaptoethanol (55mM)	0.1mM
	Pen Strep (100X)	1X
	B-27 Supplement (50X), minus vitamin A	1X
	ITS Liquid Media Supplement (100×)	1X

ME Stage (D1-D3) (version 1, Fig 4)	Activin A	50ng/mL
	CHIR99021	3μM

ME Stage (D1-D3) (version 2, Fig 5)	LDN-193189	10nM
	CHIR99021	4.5μM

UB-I Stage (D3-D5)	FGF2	200ng/ml
	TTNPB	0.1μM
	LDN-193189	30nM
	A83-01	0.2μM

UB-II Stage (D5-D7)	FGF2	200ng/ml
	TTNPB	0.1μM
	LDN-193189	30nM

Basal medium: DMEM/F12 (1:1) (1X), Invitrogen, Cat. No. 11330-032.

Supplementary Methods

Screening for optimal UB culture condition

We systematically screened the optimal UB culture condition in four different stages (Stages I-IV, Supplementary Fig. 1a). In Stage I, we tested the most updated UB culture condition from literature, Yuri *et al.*, 2017[1], in which FGF1, retinoic acid (RA), CHIR99021, and GDNF were used to allow the growth of isolated UBs *in vitro*. By repeating this condition, we confirmed that this medium supported very well the growth and branching of the UB in the first 4-5 days. However, after that, UB growth slowed down significantly, and more importantly, *Wnt11*-RFP expression was dramatically decreased (Supplementary Fig. 1b), indicating that further optimization was needed to selectively expand the *Wnt11*+ UB progenitor cells.

In Stage II, we optimized the individual medium components employed by Yuri *et al*. (FGF1, RA, CHIR99021, and GDNF). We first asked if each individual factor was necessary in the medium. For this, the factors were withdrawn from the medium individually, and the results clearly showed that all these factors were essential for maintaining the branching of UB (Supplementary Fig. 1c). Then we further optimized these factors. RA is known to be unstable in tissue culture, so we replaced it with another widely used small molecule RA substitute TTNPB. CHIR99021 was used at 1μM by Yuri *et al*., but based on our own experiences and literature, different doses of CHIR99021 often have different biological effects. So we titrated it from 1μM, 3μM, to 6μM, from which we identified 3μM to be the optimal concentration (Supplementary Fig. 1d, f). To optimize for FGF1, we tested different members from the FGF family, including FGF2, FGF4, FGF7, FGF8, FGF9, FGF10, and FGF20, from which we identified FGF9 to be superior in supporting *Wnt11*-RFP expression than FGF1 (Supplementary Fig. 1e, f). GDNF was unchanged in the medium, considering its essential role in maintaining the UB progenitor population *in vitro* and *in vivo*.

In Stage III, based on the optimized recipe consisting of FGF9, TTNPB, CHIR99021 (3μM) and GDNF, we preformed our 1st round of screening of growth factors and small molecules targeting major developmental pathways (e.g. TGF-β, BMP, Wnt, FGF, Hedgehog and Notch) and others.

The branching morphogenesis, growth rate and *Wnt11*-RFP were recorded as readouts (Supplementary Fig. f, g). From this, we identified several hits that improved either the UB growth rate or *Wnt11*-RFP, or both. Representative images were shown for LDN193189 (Supplementary Fig. 1h), A83-01 (Supplementary Fig. 1i), and R-Spondin 1 (Supplementary Fig. 1j). These individual hits were then subjected to a 2nd round of screening to test their effects in various combinations, eventually leading to the identification of the optimal UB progenitor culture medium UBCM consisting of FGF9, TTNPB, CHIR99021 (3μM), GDNF, LDN193189, A83-01, R-Spondin1, JAKI and SB202190. Representative images were shown for the combinatorial effect of JAKI and SB2020190 (Supplementary Fig. 1k), for which only marginal effects were observed when used individually (Supplementary Fig. 1f, g).

Lastly, in Stage IV, we asked whether each of the components in UBCM was essential. The factors were removed from the UBCM individually and the results indicated that all of them were necessary to achieve optimal UB organoid branching and to sustain *Wnt11*-RFP expression (Supplementary Fig. 1l).

WNT11-GFP/PAX2-mCherry dual reporter hESC line generation

CRISPR-Cas9 based genome editing was used to insert 2A-EGFP-FRT-PGK-Neo-FRT or 2A-mCherry-loxP-PGK-Neo-loxP cassette downstream of the stop codon (removed) of endogenous *WNT11* or *PAX2* gene, respectively. DNA sequences ~1Kb upstream and ~1Kb downstream of endogenous *WNT11* (upstream F: CCGGAATTCGACGTAATCATTCCACTGACC; upstream R: TACGAGCTCCTTGCAGACATAGCGCTCCAC; downstream F: CGCGTCGAC-GGCCCTGCCCTACGCCCCA; downstream R: CCCAAGCTTTGCCTGGAAACTGGA-GAGCTCCCTC) and *PAX2* (upstream F: GAAGTCGACTTTCCACCCATTAGGGGCCA; upstream R: TATGCTAGCGTGGCGGTCATAGGCAGCGG; downstream F: TATACGCGTTTAC-CGCGGGGACCACATCA; downstream R: GACGGTACCAGTAACTGCTGGAGGAAGAC) stop codon were cloned upstream and downstream of 2A-EGFP-FRT-PGK-Neo-FRT or 2A-mCherry-

loxP-PGK-Neo-loxP cassette respectively to facilitate homologous recombination. 2A-EGFP fragment was cloned from pCAS9_GFP (Addgene #44719) and the FRT-PGK-Neo-FRT cassette was cloned from pZero-FRT-Neo3R (kindly provided by Dr. Keiichiro Suzuki). 2A-mCherry-loxP-Neo-loxP fragment was cloned from Nanog-2A-mCherry plasmid (Addgene #59995). The different fragments were then cloned to pUC19 plasmid to make the complete donor plasmids for both knockin experiments. gRNA oligos for WNT11 (F: CACCGGTCCTCGCTCCTGCGTGGGG; R: AAACCCCCACGCAGGAGCGAGGACC) and PAX2 (F: CACCGATGACCGCCACTAGTTACCG; R: AAACCGGTAACTAGTGGCGGTCATC) were synthesized and cloned into the lentiCRISPR v2 plasmid (Addgene # 52961). First, both donor and gRNA plasmids for *PAX2* reporter KI were transfected into the H1 hESCs using the Lipofectamine 3000 Transfection Reagent (Invitrogen, Cat. No. L3000015). Neomycin-resistant single cell colonies were picked up manually and genotyping was performed based on PCR. PCR primers see Supplementary Table 4, results see Supplementary Fig. 4. Clones with biallelic knockin of *PAX2*-mCherry were chosen for second round screen where plasmid encoding Cre was delivered to allow the transient expression Cre, whose activities excise the loxP-flanked PGK-Neo cassette from the knockin alleles. PCR was performed to identify single cell clones in which PGK-Neo cassettes were excised from both alleles. Then the same strategy was used to knock in *WNT11* reporter based on the successful biallelic *PAX2*-mCherry knockin clones.

Gene editing in UB organoids

Gene over-expression:

Lentiviral infection was used to overexpress GFP in E11.5 mUB cells. Lentivirus was first concentrated 100x using Lenti-X Concentrator kit from Takara (Cat # 631231). Concentrated lentivirus was aliquoted and stored in -80°C before use. The lentivirus was used at 1x final concentration together with 10 μM Polybrene (Sigma-Aldrich, Cat. No. TR-1003-G) diluted in mUBCM (with 10 μM Y27632). 100 μL virus-UBCM mixture was added to the U-bottom 96-well low-attachment

plate well with single cells suspension prepared from 8-10 E11.5 mUBs. The UBs and virus were centrifuged together at 800g for 30 minutes for spinfection[10] at room temperature. After the spin-fection, the virus-UBCM mixture was removed and the infected UB cells were washed three times with PBS, then aggregated overnight and embedded in Matrigel and cultured in mUBCM in 37°C incubator following standard UB organoid culture procedures described above. 200 μg/mL G-418 (Invitrogen, Cat. # 10131027) was added to the culture to select for UB cells that have been successfully infected. The resulting UB aggregate self-organized into typical branching organoid 4-5 days after infection.

GFP knockout:

An E11.5 mUB single cell suspension from the *Rosa26*-Cas9/GFP background was used and lentiviral vectors were constructed using the lentiGuide-puro vector system (Addgene #52963) following standard protocol to make lentiviruses expressing three different gRNAs targeting GFP (gRNA sequences: F1: CACCGAAGGGCGAGGAGCTGTTCAC, R1: AAAC-GTGAACAGCTCCTCGCCCTTC; F2: CACCGCTGAAGTTCATCTGCACCAC, R2 AAAC-GTGGTGCAGATGAACTTCAC; F3: CACCGGGAGCGCACCATCTTCTTCA, R3: AAACTGAA-GAAGATGGTGCGCTCCC) with the Cas9 cutting site 100-150bp apart, or three non-targeting gRNAs as control[11]. The 100x concentrated lentivirus were used at 5x together with 10 μM Polybrene diluted in mUBCM (with 10 μM Y27632). 100 μL virus-UBCM mixture was added to the U-bottom 96-well low-attachment plate well to combine them with 10 E11.5 mUBs that have been dissociated into single cells. The UB cells and virus were centrifuged at 800 x g for 30 minutes for spin-infection. After the spin, virus-UBCM mixture was removed and fresh virus-UBCM mixture was added into the same well and the UB cells were spin-infected for another 30 minutes at 800g. Then, virus-UBCM mixture was removed and the infected UBs were washed three times with PBS, then aggregated overnight and embedded in Matrigel and cultured in mUBCM in 37°C incubator

following standard UB organoid culture procedures described above. 0.2 μg/mL puromycin was added to the medium to select for UB cells that have been successfully infected. The UB aggregate self-organized into typical branching organoid by 4-5 days post-infection.

Ret/RET knockout in mouse/human UB organoid:

Day 5 cultured mUB organoids (wildtype or any background) or stably expanded hUB organoids were dissociated into single cells following the method described above. gRNA oligos targeting mouse or human *Ret/RET* were synthesized and cloned into the lentiCRISPR v2 plasmid (Addgene # 52961) (*mRet* gRNA: F1: CACCGGAAGCTCGGCACTTCTCCAG; R1: AAACCTGGAGAAGTGCCGAGCTTCC; F2: CACCGCTGTATGTAGACCAGCCAGC; R2: AAACGCTGGCTGGTCTACATACAGC. *hRET* gRNA: F1: CACCGGTAGAGGCCCAATGCCACTG; R1: AAACCAGTGGCATTGGGCCTCTACC; F2: CACCGAAGCATCCCTCGAGAAGTAG; R2: AAACCTACTTCTCGAGGGATGCTTC). The same gRNA oligos cloned into the lentiGuide-puro vector system (Addgene #52963) that don't express the Cas9 enzyme were used as negative control. Lentiviruses with these vectors were generated following standard protocol. The 100x concentrated lentivirus were used at 2x together with 10 μM Polybrene diluted in m/hUBCM (with 10 μM Y27632). 100 μL virus-UBCM mixture was added to the U-bottom 96-well low-attachment plate well to combine them with 15,000-20,000 m/hUB single cells. The UB cells and virus were centrifuged at 800 x g for 15 minutes for spin-infection. Then, virus-UBCM mixture was removed and the infected UBs were washed three times with PBS, then aggregated overnight and embedded in Matrigel and cultured in m/hUBCM in 37°C incubator following standard UB organoid culture procedures described above. Puromycin (0.2 μg/mL for mouse and 0.3 μg/mL for human) was added to the medium two-days post-infection to select for UB cells that have been successfully infected. The UB aggregate self-organized into typical branching organoid by 2-6 days post-infection. Mouse organoids were harvested 6 days post-infection and human organoids were harvested 10-12 days post-infection for further analysis.

Human UB organoid cryopreservation

Human UB organoid were cultured until they reached the size ready for passaging. It was transferred onto 100 mm Petri dish lid and Matrigel was removed following the method described above. Organoid was then cut into 4-6 pieces using sterile needles and transferred into an Eppendorf tube. Extra medium in the tube was removed and replaced with 200 µL hUBCM with 10 µM Y27632 supplemented with DMSO at 10%. The medium and organoid pieces were then split into two cryogenic tubes for cryopreservation. To revive the organoid, the frozen cryogenic tube was thawed in 37°C water bath. Medium in the tube was removed and replaced with 50-100 µL fresh hUBCM with 10 µM Y27632. Each organoid piece was then embedded into 8 µL Matrigel and cultured in hUBCM (with 10 µM Y27632 for the first 24h) following the method described before.

Mouse Engineered Kidney Generation

The day before generating the mouse engineered kidney, 50-60k 3D cultured mNPCs was seeded per 96-well to aggregate overnight. A small piece (with 6-10 branching tips) of Day 7-10 cultured mUB organoid was manually dissected out using sterile needles (similar to passaging UB organoid as small tips mentioned above) and inserted into a microdissected hole on a 3D cultured mNPC aggregate (first, a fine dissecting tweezer was used to hold/stabilized the mNPC aggregate sphere from one side, and a sterile needle was used to pierce a hole in the center of mNPC aggregate sphere from the other side; the small piece of mUB organoid was then carefully pushed into the hole using the needle; the NPC aggregate would then slowly wrap around the inserted mUB organoid autonomously overnight; all these procedures were done in a drop (80-100 µL) of kidney reconstruction medium (APEL2 + 0.1 µM TTNPB) with 10 µM Y27632 on an inverted 100 mm plastic petri dish cap, to ensure minimal movement of the aggregate/organoid during the procedures) in kidney reconstruction medium with 10 µM Y27632 to generate a engineered kidney precursor. This engineered kidney precursor was then carefully transferred into a well of a U-bottom 96-well low-attachment plate with 100 µL kidney reconstruction medium with

10 µM Y27632, using a P200 pipette with the top 0.5-1 cm of the tip cut, and cultured in 37°C incubator (day 0). After 24 h (day 1), dead cells surrounding the precursor were removed by gently pipetting several times in the well using a P200 pipette with wide tip. The engineered kidney precursor was then transferred onto a 6-well transwell insert membrane using a wide-tip P200/P1000 pipette (depends on the size). Then 0.8-1 mL kidney reconstruction medium was added in the lower chamber of the transwell. The medium was changed every two days for a total of 7-10 days while the engineered kidney precursor maturation progressed. Then the engineered kidney was processed for further analyses.

FACS

Cells were dissociated/prepared as described above. FACS sorting was performed on a BD FACS ARIA IIIu cell sorter. Sorted cells were collected in a 1.5 mL Eppendorf tube with 500 µL 10% FBS on ice.

RNA isolation, reverse transcription and quantitative PCR

Samples were dissolved in 100 µL TRIzol (Invitrogen, Cat. No. 15596018) and kept in -80°C freezer. RNA isolation was performed using the Direct-zol RNA MicroPrep Kit (Zymo Research, Cat. No. R2062) according to the manufacturer's instructions. Reverse transcription was performed using the iScript Reverse Transcription Supermix (Bio-Rad, Cat. No. 1708841) following the manufacturer's instructions. qRT-PCR was performed using the Applied Biosystems PowerUp SYBR Green Master Mix (Thermo Fisher, Cat. No. A25777) and carried out on an Applied Biosystems Vii 7 RT-PCR system (*Life Technologies*). Validated gene-specific primers can be found in Supplementary Table 5. Fold change was calculated using the comparative CT Method (ΔΔCT Method) and *Gapdh* as housekeeping gene.

Immunofluorescence

Whole-mount staining:

Samples were fixed in 4% PFA for 45 minutes at 4°C in Eppendorf tubes with gentle shaking (UB/CD organoid, 200 µL PFA) or 10 minutes at room temperature on transwell insert membrane (kidney reconstruct, 1 mL total PFA on and below the membrane). They were then washed four times in 0.8-1 mL 1X PBS (Corning, Cat. No. 21-040-CV) for total 30 minutes at 4°C or room temperature (after the washes, kidney reconstructs on transwell membrane were cut out and transferred into Eppendorf tubes). After the washes, samples were blocked in blocking solution (0.1% PBST containing 3% BSA) for 1-2 hours at 4°C with gentle sharking, followed by primary antibody staining (primary antibodies were diluted in blocking solution) at 4°C overnight. On the second day, samples were washed three times with 800 µL 0.1% PBST for total 3 hours at 4°C with gentle sharking. Secondary antibodies diluted in blocking solution were added and samples were incubated at 4°C overnight. On the third day, samples were washed three times with 800 µL PBST for total 3 hours at 4°C with gentle sharking. Lastly, samples were mounted in mounting medium onto glass slides for imaging.

Cryo-section staining:

Samples were fixed and washed as described above. They were then transferred into a plastic mold and embedded in OCT Compound (Scigen, Cat. No. 4586K1) and froze in -80°C for 24 hours to make a cryo-block. The cryo-blocks were sectioned using Leica CM1800 Cryostat. The sectioned slides were then blocked for 30 minutes at room temperature followed by one hour of primary antibodies staining at room temperature. The slides were then washed four times with PBST for five minutes, and then secondary staining for 30 minutes. After the secondary staining, the slides were washed four times with PBST for five minutes and mounted with mounting medium.

Image quantification for UB/CD marker gene expression in the UB/CD organoids (Figure 1e, 2h, 5f, 6d, 6h, supplementary figure 6h)

Whole-mount immunostaining images for mouse UB organoids, mouse CD organoids, or human UB organoids were used for the quantification of various marker gene expression. ImageJ software (version 1.52a) was used to count positive cells. 3 different fields of view per organoid were randomly selected to count the number of positively stained cell numbers (positive for marker genes) and total cell numbers (DAPI+). Percentage was calculated by the number of cells that are positive for different UB/CD marker genes divided by the total DAPI+ cell numbers. At least 500 cells in total were counted. Error bars represent standard derivation between different field views.

Supplementary References

1. Yuri, S., Nishikawa, M., Yanagawa, N. & Jo, O. D. In Vitro Propagation and Branching Morphogenesis from Single Ureteric Bud Cells. *Stem Cell Reports* 8, 401-416, doi:10.1016/j.stemcr.2016.12.011 (2017).
2. Werth, M. et al. Transcription factor TFCP2L1 patterns cells in the mouse kidney collecting ducts. *Elife* 6, doi:10.7554/eLife.24265 (2017).
3. Taguchi, A. & Nishinakamura, R. Higher-Order Kidney Organogenesis from Pluripotent Stem Cells. *Cell Stem Cell* 21, 730-746.e736, doi:10.1016/j.stem.2017.10.011 (2017)
4. Howden, S. E. et al. Plasticity of distal nephron epithelia from human kidney organoids enables the induction of ureteric tip and stalk. *Cell Stem Cell*, doi:10.1016/j.stem.2020.12.001 (2020).
5. Uchimura, K., Wu, H., Yoshimura, Y. & Humphreys, B. D. Human Pluripotent Stem Cell-Derived Kidney Organoids with Improved Collecting Duct Maturation and Injury Modeling. *Cell Rep* 33, 108514, doi:10.1016/j.celrep.2020.108514 (2020).
6. Mae, S. I. et al. Expansion of Human iPSC-Derived Ureteric Bud Organoids with Repeated Branching Potential. *Cell Rep* 32, 107963, doi:10.1016/j.celrep.2020.107963 (2020).
7. Tsujimoto, H. et al. A Modular Differentiation System Maps Multiple Human Kidney Lineages from Pluripotent Stem Cells. *Cell Rep* 31, 107476, doi:10.1016/j.celrep.2020.03.040 (2020).
8. Mae, S. I. et al. Generation of branching ureteric bud tissues from human pluripotent stem cells. *Biochem Biophys Res Commun* 495, 954-961, doi:10.1016/j.bbrc.2017.11.105 (2018).
9. Xia, Y. et al. Directed differentiation of human pluripotent cells to ureteric bud kidney progenitor-like cells. *Nat Cell Biol* 15, 1507-1515, doi:10.1038/ncb2872 (2013).
10. Kurita, M. *et al.* In vivo reprogramming of wound-resident cells generates skin epithelial tissue. *Nature* **561**, 243-247, doi:10.1038/s41586-018-0477-4 (2018).
11. Zuo, E. *et al.* One-step generation of complete gene knockout mice and monkeys by CRISPR/Cas9-mediated gene editing with multiple sgRNAs. *Cell Res* **27**, 933-945, doi:10.1038/cr.2017.81 (2017).
