# Supplementary material for: Generation of patterned kidney organoids that recapitulate the adult kidney collecting duct system from expandable ureteric bud progenitors
Source: Nat Commun. 2021 Jun 15;12:3641. doi: 10.1038/s41467-021-23911-5 (PMC8206157; doi:10.1038/s41467-021-23911-5)
Supplement: Supplementary file 3 — Reporting Summary [file 41467_2021_23911_MOESM3_ESM.pdf]

nature research

Corresponding author(s): Zhongwei Li

Last updated by author(s): 05/11/2021

Reporting Summary

Nature Research wishes to improve the reproducibility of the work that we publish. This form provides structure for consistency and transparency in reporting. For further information on Nature Research policies, see our Editorial Policies and the Editorial Policy Checklist.

Statistics

For all statistical analyses, confirm that the following items are present in the figure legend, table legend, main text, or Methods section.

n/a	Confirmed	
☐	☒	The exact sample size (*n*) for each experimental group/condition, given as a discrete number and unit of measurement
☐	☒	A statement on whether measurements were taken from distinct samples or whether the same sample was measured repeatedly
☐	☒	The statistical test(s) used AND whether they are one- or two-sided<br>*Only common tests should be described solely by name; describe more complex techniques in the Methods section.*
☒	☐	A description of all covariates tested
☒	☐	A description of any assumptions or corrections, such as tests of normality and adjustment for multiple comparisons
☐	☒	A full description of the statistical parameters including central tendency (e.g. means) or other basic estimates (e.g. regression coefficient) AND variation (e.g. standard deviation) or associated estimates of uncertainty (e.g. confidence intervals)
☐	☒	For null hypothesis testing, the test statistic (e.g. *F*, *t*, *r*) with confidence intervals, effect sizes, degrees of freedom and *P* value noted<br>*Give P values as exact values whenever suitable.*
☒	☐	For Bayesian analysis, information on the choice of priors and Markov chain Monte Carlo settings
☒	☐	For hierarchical and complex designs, identification of the appropriate level for tests and full reporting of outcomes
☒	☐	Estimates of effect sizes (e.g. Cohen's *d*, Pearson's *r*), indicating how they were calculated

Our web collection on statistics for biologists contains articles on many of the points above.

Software and code

Policy information about availability of computer code

Data collection	no software was used
Data analysis	Prism 8 (version 8.2.1) was used for performing statistics, and Partek Flow (version 10.0.21.0411) was used for RNA-Seq data analysis, ImageJ (version 1.52a) software was used to count positive cells in immunofluorescent staining

For manuscripts utilizing custom algorithms or software that are central to the research but not yet described in published literature, software must be made available to editors and reviewers. We strongly encourage code deposition in a community repository (e.g. GitHub). See the Nature Research guidelines for submitting code & software for further information.

Data

Policy information about availability of data

All manuscripts must include a data availability statement. This statement should provide the following information, where applicable:

- Accession codes, unique identifiers, or web links for publicly available datasets
- A list of figures that have associated raw data
- A description of any restrictions on data availability

Data supporting the findings of this study are available within the paper and its supplementary information files. RNA-seq data have been submitted to Gene Expression Omnibus (GEO) with accession number GSE149109 [https://www.ncbi.nlm.nih.gov/geo/query/acc.cgi?acc=GSE149109]. GUDMAP/RBK Resources, [https://www.gudmap.org]. All other data are available from the authors upon reasonable request.

Field-specific reporting

Please select the one below that is the best fit for your research. If you are not sure, read the appropriate sections before making your selection.

- [x] Life sciences
- [ ] Behavioural & social sciences
- [ ] Ecological, evolutionary & environmental sciences

For a reference copy of the document with all sections, see nature.com/documents/nr-reporting-summary-flat.pdf

Life sciences study design

All studies must disclose on these points even when the disclosure is negative.

Sample size	All samples evaluated at the level of RNAseq were performed in duplicate or triplicate to facilitate a statistical evaluation of differential expression. All experiments reported in this study were repeated at least three independent times. Representative images (micrographs) were derived from at least 3 organoids that all showed similar results. Figure 1e, 2h, 5f, 6d, 6h, supplementary figure 6h data were collected from 3 different fields of view per organoid. qRT-PCR data were performed in triplicate to facilitate statistical evaluation. Sample size/ replication and other statistics of generating engineered kidneys and collect duct organoids are showing in Figure 3e and Supplementary Figure 3o.
Data exclusions	No data were excluded
Replication	All experiments reported in this study were repeated at least three independent times. All samples evaluated at the level of RNAseq were performed in duplicate or triplicate and the sequencing was all successful. Representative images (micrographs) were derived from at least 3 organoids that all showed similar results. Figure 1e, 2h, 5f, 6d, 6h, supplementary figure 6h data were collected from 3 different fields of view per organoid. qRT-PCR data were performed in triplicate and were mostly successful with a few failure due to technique issues. The number of replication and successful rate of generating engineered kidneys and collect duct organoids are showing in Figure 3e and Supplementary Figure 3o.
Randomization	Samples were allocated into experiment group randomly
Blinding	The investigators were blinded to group allocation during data collection and analysis.

Reporting for specific materials, systems and methods

We require information from authors about some types of materials, experimental systems and methods used in many studies. Here, indicate whether each material, system or method listed is relevant to your study. If you are not sure if a list item applies to your research, read the appropriate section before selecting a response.

Materials & experimental systems

n/a	Involved in the study	
☐	☒	Antibodies
☐	☒	Eukaryotic cell lines
☒	☐	Palaeontology and archaeology
☐	☒	Animals and other organisms
☒	☐	Human research participants
☒	☐	Clinical data
☒	☐	Dual use research of concern

Methods

n/a	Involved in the study	
☒	☐	ChIP-seq
☐	☒	Flow cytometry
☒	☐	MRI-based neuroimaging

Antibodies

Antibodies used	anti-KRT8 DSHB Cat.# TROMA-I 1:50; GATA3 R&D Systems Cat.# AF2605 1:1000; SOX9 Abcam Cat.# 185230 1:1000; RET (mouse) Cell Signaling Technology Cat.# 3223S 1:300; RET (human) R&D Systems Cat.# AF1485 1:200; PAX2 Covance Cat.# PRB-276P 1:1000; PAX8 Proteintech Cat.# 10336-1-AP 1:1000; ETV5 Abcam Cat.# Ab102010 1:300; CDH1 BD Cat.# 610181 1:1000; AQP2 Santa Cruz Cat.# sc-47710 1:300; AQP3 Abcam Cat.# ab125219 1:300; FOXI1 Novus Biologicals Cat.# NB300-926 1:300; ATP6V1B1 Abcam Cat.# ab192612 1:300; TFCP2L1 R&D Systems Cat.# AF5726 1:300; KIT Cell Signaling Cat.# 3074 1:300; PE-CD117(c-kit) Biolegend Cat.# 313204 1:200; SIX2 Proteintech Cat.# 11562-1-AP 1:1000; PODXL (mouse) R&D Systems Cat.# MAB1556 1:500; WT1 Abcam Cat.# ab89901 1:1000; LTL Vector laboratories Cat.# B-1325 1:500; T R&D Systemss Cat.# AF2085 1:300; Donkey anti-Goat, Alexa Fluor 568, Invitrogen, Cat. # A-11057;
Validation	All primary antibodies were validated as indicated in the manufacturers' website. We further validated them by mouse or human fetal kidney tissue section IF-staining. anti-KRT8 DSHB validated in both human and mouse; GATA3 R&D Systems validated in both human and mouse; SOX9 Abcam validated in both human and mouse; RET (mouse) Cell Signaling Technology validated in mouse; RET (human) R&D Systems validated in human; PAX2 Covance validated in both human and mouse; PAX8 Proteintech validated in both human and mouse; ETV5 Abcam validated in mouse; CDH1 BD validated in both human and mouse; AQP2 Santa Cruz validated in

mouse; AQP3 Abcam validated in both human and mouse; FOXI1 Novus Biologicals validated in both human and mouse; ATP6V1B1 Abcam validated in both human and mouse; TFCP2L1 R&D Systems validated in mouse; KIT Cell Signaling validated in both human and mouse; PE-CD117(c-kit) Biolegend validated in human; SIX2 Proteintech validated in both human and mouse; PODXL (mouse) R&D Systems validated in mouse; WT1 Abcam validated in both human and mouse; LTL Vector laboratories validated in both human and mouse; T R&D Systemss validated in human.

Eukaryotic cell lines

Policy information about cell lines

Cell line source(s)	H1 (WA01) cell line was obtained from WiCell; Sox9-GFP iPSC line was shared from NIH. Dual reporter H1 hESC line, 3D cultured NPC, and all mouse and human UB organoids were development in our previous study (Li et al. 2016) or this study in our lab.
Authentication	hPSC lines were authenticated regularly by qPCR and IF for various pluripotency gene expression. 3D cultured NPC and all mouse and human UB organoids were authenticated regularly by qPCR and IF for various NPC/UB marker gene expression.
Mycoplasma contamination	All cell lines were tested negative for mycoplasma contamination.
Commonly misidentified lines (See ICLAC register)	No misidentified line was used in the manuscript.

Animals and other organisms

Policy information about studies involving animals; ARRIVE guidelines recommended for reporting animal research

Laboratory animals	Female Swiss Webster mice were purchased from Taconic Biosciences (Model # SW-F, MPF 4 weeks). 2-10 months-old Male Wnt11-RFP mice (JAX # 018683), Hoxb7-Venus mice (JAX # 016252), Rosa26-Cas9/GFP mice (JAX #026179), Sox9-GFP mice (kindly shared from Dr. Haruhiko Akiyama) were used for mating with female Swiss Webster mice.<br>Housing condition: Mice were maintained under standard conditions (standard diet and water) at 23 °C and ~60% humidity with 12h light and 12h dark cycles.
Wild animals	None were used
Field-collected samples	None were used
Ethics oversight	All animal work was performed under Institutional Animal Care and Use Committee approval (USC IACUC Protocol # 20829).

Note that full information on the approval of the study protocol must also be provided in the manuscript.

Flow Cytometry

Plots

Confirm that:

- [x] The axis labels state the marker and fluorochrome used (e.g. CD4-FITC).
- [x] The axis scales are clearly visible. Include numbers along axes only for bottom left plot of group (a 'group' is an analysis of identical markers).
- [x] All plots are contour plots with outliers or pseudocolor plots.
- [x] A numerical value for number of cells or percentage (with statistics) is provided.

Methodology

Sample preparation	Embryonic kidneys or organoids were dissociated into single cells with Accumax, and then resuspended with FACS medium (2% FBS plus PBS), and then cell solution went through 40um cell strainer (Greiner bio-one, Cat. No. 542040), next the samples were ready for FACS analysis.
Instrument	BD FACSAria IIIu instrument was used for sorting (100 uM nozzle was used and 20 psi pressure was applied).
Software	FACS Vantage (Aria II and FACS Jazz).
Cell population abundance	We determined the relevant cell population based on cell surface markers after gating on the RET/Pax2-mCherry/Sox9-GFP/ KIT positive cell population from human fetal kidney tissues or differentiated hPSCs population. Each cell population is presented as % of whole human fetal kidney cells or differentiated hPSCs; RET+ primary human UPCs (1-2%), Pax2-mCherry+ UPC precursor cells (~13.5%), Sox9-GFP+ UPC precursor cells (60.2%), KIT+ UPC-like cells (36.1-43.9%).
Gating strategy	Gating strategies are clearly described in the figure legends. Basically, live cells were gated with FCS and SSC and subsequently gated with various markers as indicated in the figures.

- [x] Tick this box to confirm that a figure exemplifying the gating strategy is provided in the Supplementary Information.
