# Supplementary material for: Generation of patterned kidney organoids that recapitulate the adult kidney collecting duct system from expandable ureteric bud progenitors
Source: Nat Commun. 2021 Jun 15;12:3641. doi: 10.1038/s41467-021-23911-5 (PMC8206157; doi:10.1038/s41467-021-23911-5)
Supplement: Supplementary file 4 — Description of Additional Supplementary Files [file 41467_2021_23911_MOESM4_ESM.docx]

Description of Additional Supplementary Files

Title: Supplementary Data 1

Description: RNA-seq TMM values.

Title: Supplementary Data

Description: 4-fold change gene list from RNA-seq (tip vs trunk)
